# A hybrid KF–GAIN framework for dynamic state estimation in islanded microgrids under missing PMU data and line impedance uncertainty

**DOI:** 10.1038/s41598-026-51565-0

**Published:** 2026-05-22

**Authors:** Mohammad Reza Masoudi, Mohammad Mohammadi, Behrooz Zaker, Dariush Keihan Asl, Majid Mostafanezhad

**Affiliations:** 1 Department of Power and Control Engineering, School of Electrical and Computer Engineering, Shiraz University, Shiraz, Iran; 2 Department of Electrical Engineering, Persian Gulf University, Bushehr, Iran; 3 Department of Electrical and Computer Engineering, University of Toronto, Toronto, ON M5S 3G4 Canada

**Keywords:** Islanded microgrid, Dynamic state estimation, Kalman filter, Deep learning, Probabilistic line impedance, Missing data, Energy science and technology, Engineering, Mathematics and computing

## Abstract

The similar time constants of voltage source inverter-based distributed generation units and other components in islanded microgrids (IMGs) pose significant challenges for dynamic state estimation (DSE). To address these issues, particularly under conditions of missing measurement data and uncertainties in line impedances, this paper proposes a linear hybrid framework that combines the Kalman Filter (KF) with a deep learning model called the generative adversarial imputation network (GAIN) for DSE in IMGs. GAIN robustly imputes missing data while preserving the statistical properties of the original dataset, aided by a hint mechanism that guides the generator to produce values closely following the true data distribution. The proposed KF-GAIN model is evaluated on an islanded IEEE 33-bus test system equipped with distributed generation units and phasor measurement units (PMUs), incorporating temperature-dependent line impedance variations modeled via Monte Carlo simulation. The IMG is first simulated in MATLAB/Simulink; subsequently, the measurement data are then transferred to Python for missing data imputation using GAIN, followed by linear DSE via the KF. To assess GAIN’s performance, autoencoder (AE) and variational autoencoder (VAE) models are employed as baseline methods. Hyperparameters (GAIN, AE, VAE) were selected by grid search; robustness was assessed via sensitivity to single and pairwise PMU outputs. GAIN achieves a significantly lower imputation error (0.02705) compared to AE (0.35872) and VAE (0.20344), demonstrating superior accuracy. Furthermore, the proposed framework maintains high estimation accuracy, with average errors of 0.46% and 0.72% for 20% and 40% missing data, respectively, across multiple scenarios.

## Introduction

### Motivation and aims

Distribution-level power systems are undergoing a transformation from traditional passive configurations to more active and decentralized structures, which are increasingly organized into smaller, more manageable subsystems known as microgrids^1^. Microgrids can operate in two modes—grid-connected and islanded—both of which contribute to improving the reliability and flexibility of the overall power system. In the grid-connected mode, microgrids can support the main grid by supplying auxiliary power, while in the islanded mode, they are capable of operating independently from the main grid^2^. An islanded microgrid (IMG) operates independently, either intentionally, for purposes such as maintenance or economic benefits, or unintentionally due to faults in the main grid, typically triggered by opening a switch. IMGs, in particular, offer a reliable and environmentally friendly energy solution for remote and off-grid locations. Their successful operation depends on the effective coordination of distributed generation (DG) resources and intermittent loads, while maintaining system stability and resilience.

Energy management systems (EMSs) can be employed to enhance the operational efficiency of IMGs by regulating voltage and frequency, while also enabling real-time monitoring, control, and optimization of DG units. State estimation (SE) constitutes a vital component of the EMS, enabling system operators to assess the system’s state variables, even in the presence of faulty or missing measurements^3^. The main task of SE is to eliminate errors in the measurements^4^, enabling the accurate estimation of key parameters such as bus voltage magnitudes and phase angles based on available real-time data. Precise SE not only improves the reliability and stability of IMG operations but also forms the basis for advanced control strategies, fault detection mechanisms, and informed decision-making processes within the EMS.

In IMGs, particularly under dynamic conditions, SE is essential for maintaining system observability, enabling reliable control, and ensuring efficient operation despite limited measurements and high variability. Accordingly, dynamic state estimation (DSE) is well-suited for accurately capturing the evolving system states, which is critical for the effective control and protection of IMGs^5^. The application of DSE techniques is essential for the operation of an IMG, where the system relies entirely on its own resources to meet the load demand. DSE in IMGs aims to accurately determine system states despite limited data and uncertainties from disturbances, ensuring reliable control and system stability. Moreover, DSE is generally favored over static state estimation (SSE) in scenarios requiring real-time or time-varying insights, as it offers a more detailed understanding of system behavior and supports more informed decision-making in dynamic environments^6^. This makes DSE a vital tool for enhancing the resilience and responsiveness of modern IMG operations.

Measurement devices such as phasor measurement units (PMUs) are strategically deployed within IMG environments to monitor and record key parameters, including voltage, current, and active/reactive power, which are then transmitted to the EMS integration center^7^. However, these measurements are often affected by noise and occasional device or communication failures, potentially leading to data loss. Accurately estimating states presents significant challenges due to missing data and uncertainties in system parameters. These issues can lead to inaccurate DSE, ultimately affecting the stability and reliability of the IMG. Consequently, developing an effective framework for IMG operation that accounts for missing data and addresses these associated challenges is of critical importance. Thus, implementing robust DSE techniques capable of managing missing or faulty data is critical to ensure reliable operation and stability in IMGs.

### Literature review

Over the past decade, numerous studies have been conducted on SE in microgrids using a variety of filtering techniques. Among the most commonly employed methods are Kalman filters (KFs), extended Kalman filters (EKFs), unscented Kalman filters (UKFs), particle filter (PF), and weighted least squares (WLSs).

The KF algorithm is often considered the optimal choice for SE in microgrids with linear models. In this context, the KF is employed as a state estimator for a linearized dynamic model of the microgrids. Due to its recursive nature, the KF requires only the previous SE and the current measurements to compute the current SE, unlike many other estimators that rely on storing the entire history of measurements and estimates^8^.

The EKF has been widely used for SE in control systems^9^. However, its reliance on the Jacobian matrix, used to linearize nonlinear functions, introduces several challenges. Calculating these derivatives can be complex and computationally demanding, especially in large-scale systems. Furthermore, if the time-step intervals are not sufficiently small, the linearization process can lead to filter instability. As system complexity increases, the EKF may struggle to converge or become difficult to implement. Additionally, the need to compute partial derivatives at every step makes the EKF an iterative and computationally expensive algorithm. The UKF has emerged as a promising alternative to overcome the limitations of the EKF, as discussed in^10,11^.

Unlike the EKF, the UKF employs a deterministic sampling technique, representing the state distribution with a carefully selected set of sigma points that capture the mean and covariance accurately. This approach preserves system nonlinearity without requiring Jacobian computations. However, the UKF may exhibit degraded performance when the process or measurement noise deviates from Gaussian distributions^12^.

The PF is based on a Bayesian SE (BSE). In BSE, creating a posterior probability density function based on input measurements and other information, including the theoretical state model, initial system SE, and probability distribution of uncertainty in both state and observation models, is essential. This approach is known as a Bayesian perspective in DSE. The basis of this method is the use of particles, which during the algorithm process, provide an estimate of the posterior distribution, making it possible to estimate the desired parameter. Table 1 provides a comprehensive comparison of commonly used SE methods, highlighting their respective advantages, limitations, and suitability for DSE in IMGs^13^.

**Table 1:** Comprehensive comparison of SE methods.

Category	SE Method	Advantages	Disadvantages	Suitability for IMG-DSE
Classical Statistical	WLS	Simple implementation; low computational cost	Sensitive to bad/missing data; static formulation	Limited (static only)
	Robust WLS (Huber, LAV)	Improved bad-data suppression	Iterative; higher computational cost	Moderate
Bayesian/Linear Filtering	KF	Optimal for linear Gaussian systems; recursive; low computational cost	Requires accurate system model; linear assumption	Highly suitable (linearized IMG models)
Bayesian/Nonlinear Filtering	EKF	Handles nonlinear systems; widely used	Linearization errors; possible divergence	Moderate
	UKF	Better nonlinear approximation than EKF	Higher computational cost; numerical instability risk	Moderate
	PF	Handles highly nonlinear & non-Gaussian systems	Very high computational cost; particle degeneracy	Limited (real-time constraints)
Robust/Uncertainty-aware	H∞ Filter	Robust against model uncertainty	More conservative; tuning complexity	Moderate
	Moving Horizon Estimation (MHE)	Handles constraints; robust to noise	High computational burden (optimization-based)	Limited (real-time heavy)
Machine Learning-Based	ANN-based SE	Model-free; captures nonlinear mapping	Requires large dataset; poor extrapolation	Limited physical interpretability
	Autoencoder (AE)	Effective for feature extraction	Weak generative capability for missing data	Used as baseline
	Variational Autoencoder (VAE)	Probabilistic latent modeling	Blurred reconstruction; training instability	Used as baseline
	GAN-based SE	Strong generative ability	Training instability; mode collapse	Promising but complex
Hybrid ML + Statistical	KF + GAIN (Proposed)	Robust missing data handling; preserves statistical distribution; low computational linear DSE; physically consistent	Requires offline training of GAIN	Highly suitable

After presenting the overview of filtering techniques, this paper reviews existing studies that have applied these methods to SE in microgrids. For example, in^14^, a learning-based DSE is proposed for networked microgrids with unknown dynamics. By combining neural-ordinary-differential-equations and KFs, it accurately tracks system states even with limited data and model uncertainties, showing improved performance in simulations. In^15^, a KF-based method is proposed to jointly estimate states and unknown inputs in microgrids using a physics-based linear model, offering improved accuracy and robustness over traditional approaches. A UKF-based method for SE in an IMG, supplying an electric vehicle charging station, is proposed in^16^, where a reduced nonlinear model is used to estimate system states accurately under disturbances and noisy inputs, with simulations validating its robustness and precision. A linear SE model for hybrid AC/DC microgrids, focusing on voltage source inverters (VSIs), is proposed in^17^. An adaptive KF is used to enhance resilience and reduce computational burden, demonstrating comparable accuracy to nonlinear WLS methods with improved efficiency.

A machine learning-based SE method is proposed in^18^ for unobservable, AC microgrids with heteroscedastic noise. It avoids pseudo-measurements, handles sensor issues, and outperforms traditional WLS by using limited simulations and recursive noise modeling. A SE method for IMGs is proposed in^19^, which enhances the traditional WLS approach by integrating droop equations of DGs. Tested on a large-scale 906-bus system, the method shows improved accuracy and robustness against initialization and measurement noise compared to conventional WLS. A non-iterative SSE method is proposed in^20^ for microgrids with DGs, using a linear measurement model based on optimally placed PMUs that provide complex branch currents and voltages. By avoiding Jacobian computations and integrating M-estimator-based bad data detection, the method enhances efficiency and robustness. An adaptive nonlinear control strategy is proposed in^21^ to address the instability of converters in microgrids with constant power loads. The method integrates an adaptive passivity-based controller with an adaptive EKF to enhance stability, estimate unknown loads without extra sensors, and improve dynamic response under uncertainties.

A novel application of the unscented PF is presented in^22^ for DSE of a doubly fed induction generator operating as part of a microgrid within a multi-microgrid system. Utilizing only local PMU measurements, the proposed method demonstrates superior accuracy and robustness compared to conventional PF and UKF approaches, particularly under system disturbances, while effectively addressing particle degeneration issues. A nonlinear PF approach for DSE in droop-controlled IMGs is introduced in^23^. This method enhances the robustness of PFs by enabling them to process noisy measurements from various probability distributions. Although PF is a good state estimator for nonlinear systems, there is a high computational complexity in implementing this algorithm, resulting in high computational costs.

In^24^, a DSE-based method is proposed to improve fault detection in IMGs with converter-interfaced generation penetration. By using time-domain measurements and dynamic models, the approach enhances internal fault detection and improves frequency control using local data. The DSE problem for an explicit model of IMGs with fading measurements is addressed in^25^, where a fading measurement model is proposed along with a recursive estimator to minimize estimation errors, better reflecting real-world conditions. A supply adequacy-based optimal zone clustering algorithm for IMGs is reported in^26^, taking into account the dynamic performance of distributed SE units. A comprehensive modeling approach for DSE in IMG structures is addressed in^27^, enabling the capture of various dynamic phenomena related to the electrical grid and generator units. In^28^, DSE for a microgrid is conducted using the UKF and EKF with a classical generator model. Additionally, a scheme is developed to incorporate delayed data in KF estimation, enabling the simulation of data loss and communication delays in the microgrid.

In^29^, a centralized dynamic-algebraic SE method is proposed for microgrids using PMU data to estimate both algebraic and dynamic states. A novel PMU placement strategy ensures full observability, while least absolute value estimation handles bad data and feeds pseudo-measurements to a UKF for accurate DSE. Simulation results confirm the method’s effectiveness for nonlinear generator and turbine models. In^30^, a secure distributed SE method is proposed to detect and isolate faulty nodes in networked microgrids. The method uses a trust-based diffusion algorithm, where each microgrid agent gives more weight to trusted neighbors and ignores untrusted ones. This helps form secure clusters in real time and improves the system’s ability to handle data integrity and security issues. In^31^, a PMU-based robust SE method is proposed for integrated microgrids. The problem is formulated as a quadratic program and solved in a decentralized way to support autonomous microgrid operation and preserve data privacy. Each microgrid detects bad data locally and shares boundary-related objectives with the utility grid for coordinated optimization.

In^32^, a robust IoT-based framework is proposed for real-time SE of unstable microgrids. Using a delay-universal error correction code and iterative estimation, the method improves reliability over wireless networks and outperforms traditional coding schemes in tracking accuracy. Additionally, a distributed SE method is developed in^33^ for IMGs with nonlinear uncertainties and time delays, where observers use partial measurements and consensus mechanisms to ensure stable and accurate SE.

PMUs play a vital role in the real-time monitoring of electrical parameters and are widely used in microgrids. However, one of the major challenges associated with PMUs, particularly in IMGs, is the unavailability of measurement data^34^. These units typically measure voltage and current in the direct and quadrature (dq) reference frame. Nevertheless, due to various factors such as communication delays, network outages, or device malfunctions, the measured data may become temporarily or permanently inaccessible. To enhance resilience, two general approaches can be adopted in data-driven methods. The first approach involves modifying the artificial intelligence model’s structure to enable its performance under conditions of incomplete observability. For instance, in^35,36^, changes in decision tree structures have been used to make the model more robust against data loss. Another approach is data recovery or replacement, where lost data is compensated for by existing data or synthetic data. For example, in^37^, an attempt has been made to recover measurement information by estimating missing values. Today, with the advancements in deep learning, it is possible to create models that perform better in data recovery. Generative adversarial network (GAN) is a framework that can be used in microgrids to deal with the missing data from PMUs for security reasons, as shown in^38^. The approach leverages GANs to effectively recover missing data, without relying on PMU placement or network topology. However, this structure still faces challenges, such as dealing with changes in all data (both missed and retained data).

Traditional model-based approaches, such as the EKF^39^, ensure synchronized dynamic states based on generator models, while integrating compressive sensing (KF-ModCS) enables state recovery under sparse measurements^40^. Advancements in handling nonlinearity include sparse regression combined with the UKF^41^. Learning-based DSE, specifically long short-term memory (LSTM) networks, has demonstrated effectiveness in mitigating the impact of fluctuating renewable generation^42^, and robust hybrid state estimation (RHSE) fuses PMU and supervisory control and data acquisition (SCADA) data to counter bounded measurement uncertainties^43^.

However, a significant architectural gap remains: most contemporary methods treat dynamic estimation and missing data recovery as siloed tasks or fail to seamlessly integrate learning-based imputation into the core model-based estimator. The proposed KF-GAIN framework resolves this by unifying GAN based missing data imputation directly with KF-based DSE. Furthermore, the inclusion of probabilistic modeling for line impedance uncertainties enhances the robustness and accuracy of the resulting SE for IMGs. To explicitly situate the contribution of the proposed method, Table 2 provides a comprehensive comparison detailing the architecture, core components (model-based vs. learning-based), handling of missing data, DSE capability, and application domain of contemporary literature alongside the unique unified structure of the KF-GAIN framework.

**Table 2:** Comparative overview of SE frameworks highlighting the integration of dynamic models and data imputation.

Ref.	Method	Model-based estimator	Learning-based	Missing data recovery	DSE	Microgrid	Notes
^31^	Distributed microgrid observer	✓	✗	Limited	✓	✓	Local bad data detection + coordination
^32^	IoT-based SE	✓	✗	Limited	✓	✓	Real-time estimation under network delays
^33^	PMU-based DSE	✓	✗	✗	✓	✓	Real-time monitoring; missing measurements not handled
^34–36^	Data-driven recovery	✗	✓	✓	Partial	✓	Learning-based recovery of missing PMU data
^37^	GAN-based recovery	✗	✓	✓	✗	✓	Missing PMU data recovery without topology
^39^	EKF-based DSE	✓	✗	✗	✓	Partial	Synchronized dynamic states from generator models
^40^	KF + Compressive Sensing	✓	✗	✓	✓	Partial	Sparse/intermittent measurements in distribution grid
^41^	Sparse regression + UKF	✓	Partial	Limited	✓	Partial	Nonlinear systems; model identification + UKF
^42^	LSTM-based DSE	✗	✓	Limited	✓	Partial	Renewable integration; limited measurements
^43^	RHSE	✓	✗	Limited	✓	Partial	Robust against bounded data uncertainties
Proposed	KF-GAIN	✓	✓ (GAIN)	✓	✓	✓	Unified: GAIN + KF + probabilistic line impedance

Although extensive research has been conducted on SE in microgrids, the specific issue of DSE in IMGs, particularly under conditions of missing measurement data, remains largely unexplored. Furthermore, there is a notable lack of comprehensive probabilistic modeling of line impedance variations. Incorporating uncertainties and random fluctuations in line impedances is crucial for enhancing the accuracy and robustness of DSE in IMGs, thereby providing a more realistic representation of system behavior under practical operating conditions. Consequently, this paper aims to address these gaps by developing a framework for DSE in IMGs that incorporates missing data imputation and probabilistic line impedance modeling.

### Contributions and aims

This paper presents a linear hybrid framework for DSE in IMGs, which integrates the KF with a deep learning model known as the generative adversarial imputation network (GAIN)^44^. In contrast to existing KF-based and learning-assisted approaches, the proposed KF–GAIN framework integrates a GAIN directly within the Kalman filtering loop, rather than treating SE and data imputation as separate processes. In addition, it incorporates probabilistic modeling of line impedance uncertainties, resulting in a unified DSE architecture that jointly addresses missing data recovery and parameter uncertainty, which are not jointly considered in most prior studies. The proposed hybrid GAIN–KF framework is structured for real-time deployment via distinct offline and online workflows. The offline stage is strictly limited to the once-only training of the GAIN network. During real-time execution, the system processes measurements sequentially: the GAIN module is invoked only when PMU data are missing, reconstructing the required inputs using the instantaneous statistical distribution of available sensors via a single, fast forward pass. If data are complete, GAIN is bypassed. The resulting (raw or imputed) vector is fed to the KF for SE. This ensures computational overhead is incurred only upon failure. The KF enables real-time, low-overhead estimation; GAIN preprocesses measurements to withstand missing data and noise, making the framework fast and robust. This combined approach addresses challenges arising from probabilistic line impedances and partial PMU data loss. These challenges reflect real-world conditions more accurately, especially in systems where DGs are interfaced via VSIs. In particular, temperature-induced uncertainties in line impedances are modeled probabilistically using Monte Carlo simulations (MCSs), allowing a realistic representation of operating conditions. The proposed method enhances both the accuracy and robustness of DSE under such uncertain and incomplete data scenarios. Missing measurements, caused by sensor faults, communication failures, or environmental noise, can significantly degrade DSE performance and impair critical decision-making during emergencies. To mitigate this, the framework incorporates GAIN as a deep learning-based data imputation strategy. GAIN reconstructs missing data through an adversarial process involving a generator and a discriminator, improving estimation accuracy and overall system reliability. A key innovation of GAIN, compared to conventional GANs, is its hint mechanism, which provides the discriminator with partial information about observed data locations. This enables the generator to better learn the underlying data distribution, yielding high-fidelity reconstructions that preserve the statistical characteristics of the original dataset.

To assess the effectiveness of the proposed GAIN-based framework for missing data imputation, two widely recognized deep learning models, autoencoder (AE) and variational autoencoder (VAE), are employed as baseline methods^45^. Model hyperparameters (GAIN, AE, VAE) were chosen by grid search on validation data; robustness is examined via sensitivity analysis on single and pairwise PMU outputs. Although GAIN does not utilize a conventional encoder–decoder structure like AE and VAE, it shares the fundamental objective of reconstructing incomplete data. AE and VAE are well-established for capturing complex nonlinear dependencies among features, making them suitable benchmarks for evaluating imputation performance. However, GAIN introduces a fundamentally different architecture by incorporating a generative-discriminative adversarial structure. This allows it to more accurately model the distribution of missing data and generate realistic, context-aware imputations. The use of adversarial learning in this context is a key innovation that distinguishes GAIN from traditional autoencoding approaches.

From an implementation perspective, the IMG is first modeled in MATLAB/Simulink using the nonlinear dynamic behavior of DG units and VSIs, with PMU-based data acquisition. Following data collection, a linear hybrid framework combining the KF and GAIN is implemented in Python to perform DSE under incomplete data conditions. Decoupled simulation–then–estimation (Simulink → Python) is a key feature, not an implementation detail. It cleanly separates high-fidelity modeling from low-cost real-time estimation, enabling independent scaling, robust testing (PMU loss, disturbances), and plug-and-play replacement of plant models or estimators. Finally, the effectiveness of the proposed approach is validated through simulations on an islanded IEEE 33-bus test system, where five buses are equipped with both DG units and PMUs^26^. The main contributions of this paper are summarized as follows:

1. Hybrid KF-GAIN framework for robust DSE: A novel linear hybrid framework is proposed that integrates the KF with the GAIN model, combining model-based estimation with data-driven imputation^46^. This synergy enables reliable DSE performance under partial observability and incomplete data scenarios in IMGs.
2. Explicit modeling of uncertainty in system parameters: The framework incorporates probabilistic variations in line impedance due to ambient temperature and accounts for measurement noise in PMU data, improving estimation robustness in realistic and dynamically changing environments.
3. Advanced data imputation using GAIN: GAIN is applied to reconstruct missing PMU measurements by leveraging adversarial learning and a hint mechanism, outperforming baseline models such as AE and VAE in capturing the underlying data distribution without relying on encoder–decoder structures.
4. Modular linear DSE pipeline with simulation-data decoupling: The nonlinear dynamics of IMG components are simulated offline in MATLAB/Simulink to produce PMU measurements. These measurements are then used in a purely linear estimation stage via KF, enabling computational simplicity while maintaining model fidelity.

The remainder of this paper is organized as follows. Section 2 discusses the mathematical modeling of IMG components, emphasizing their dynamic behavior. Section 3 introduces the proposed hybrid KF-GAIN framework for DSE. Section 4 presents the simulation results, and Sect. 5 concludes the paper.

## Mathematical formulation

**Fig. 1:**
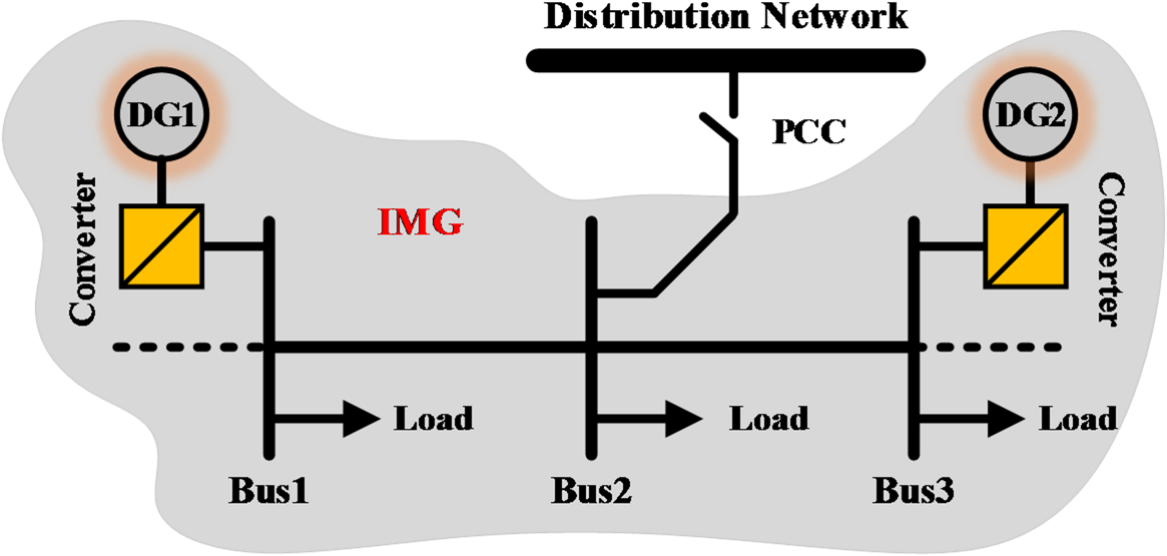
A general overview of an IMG.

DG units are responsible for supplying both active and reactive power demands in an IMG, while ensuring voltage and frequency stability^26^. As shown in Fig. 1, DG units are typically interfaced with the IMG through VSIs. These inverters facilitate integration but lack the inherent physical inertia of conventional generators. In an IMG, each DG operates at its own angular frequency, resulting in multiple distinct *dq* reference frames. Therefore, all state variables associated with DGs, transmission lines, and loads must be transformed into a common reference frame, typically that of the original DG^23^. This section presents the detailed VSI modeling and dynamic modeling of an IMG, with a particular emphasis on the requirements for DSE and the impact of line impedance uncertainty. These models serve as a foundation for the DSE implementation discussed in Sect. 3.

### Converter modeling

**Fig. 2:**
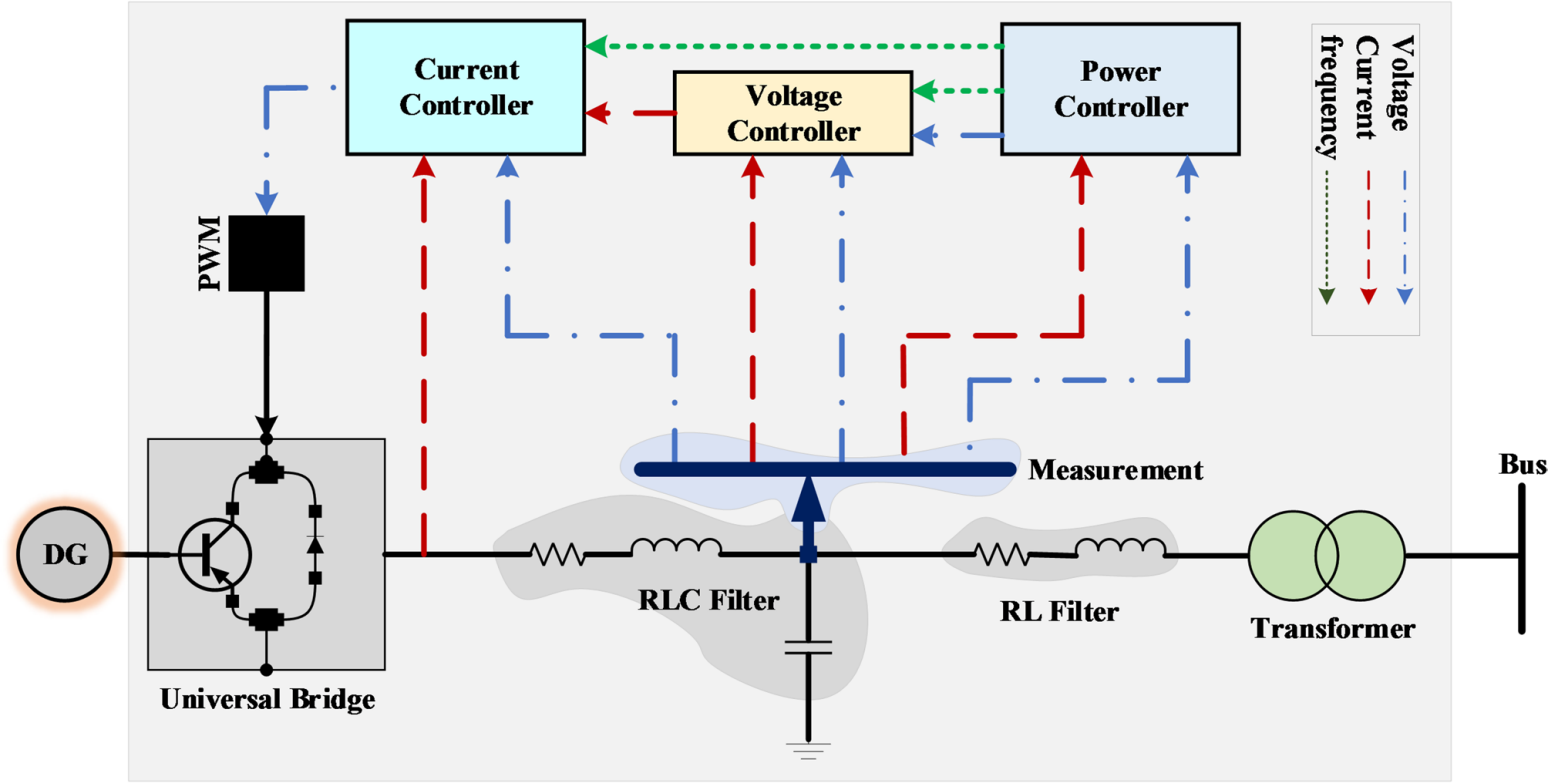
Block diagram of a converter.

Figure 2 illustrates a detailed block diagram of the VSI connected to the IMG. The VSI controller consists of hierarchical control loops, including power, voltage, and current control loops. Other key components of the system include a DC-to-AC conversion unit, RLC and RL filters to mitigate harmonic distortion, and a transformer to adjust the voltage to the appropriate range. Additionally, pulse width modulation (PWM) is employed to generate an efficient and accurate AC waveform^47^.

**Fig. 3:**
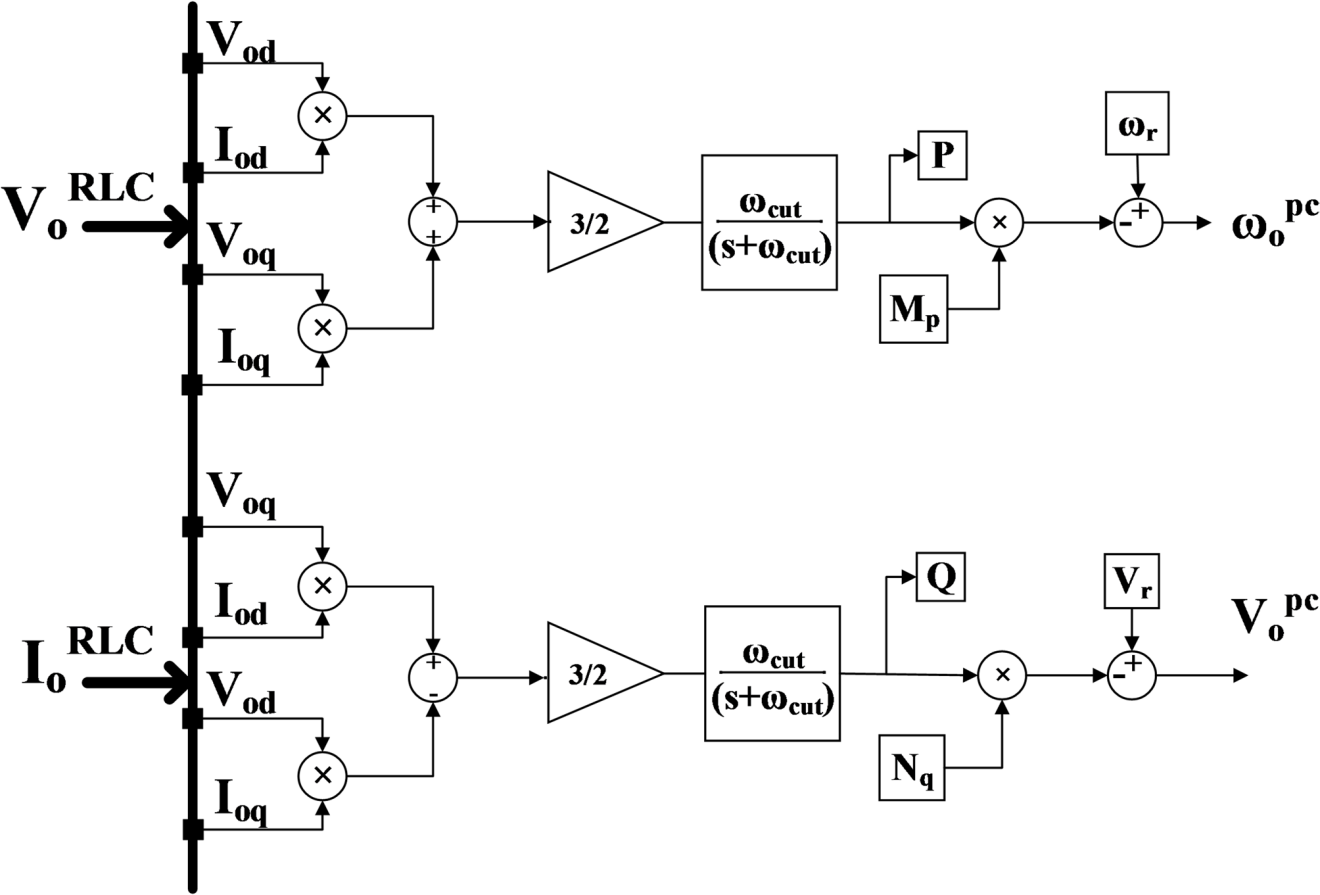
Block diagram of power controller.

The controller of the VSI is typically divided into three main components: the power controller, the voltage controller, and the current controller. As illustrated in Fig. 3, the power controller calculates the instantaneous active and reactive power using the voltage ($\:{V}_{o}^{RLC}$) and current ($\:{I}_{o}^{RLC}$) measured at the output of the RLC filter. The power control strategy is based on droop control, which enables proportional power sharing among DG units according to their rated capacities. The instantaneous power signals are passed through low-pass filters to extract the fundamental components of active power (*P*) and reactive power (*Q*), where $\:{\omega\:}_{cut}$ denotes the cutoff frequency of the filters.

**Fig. 4:**
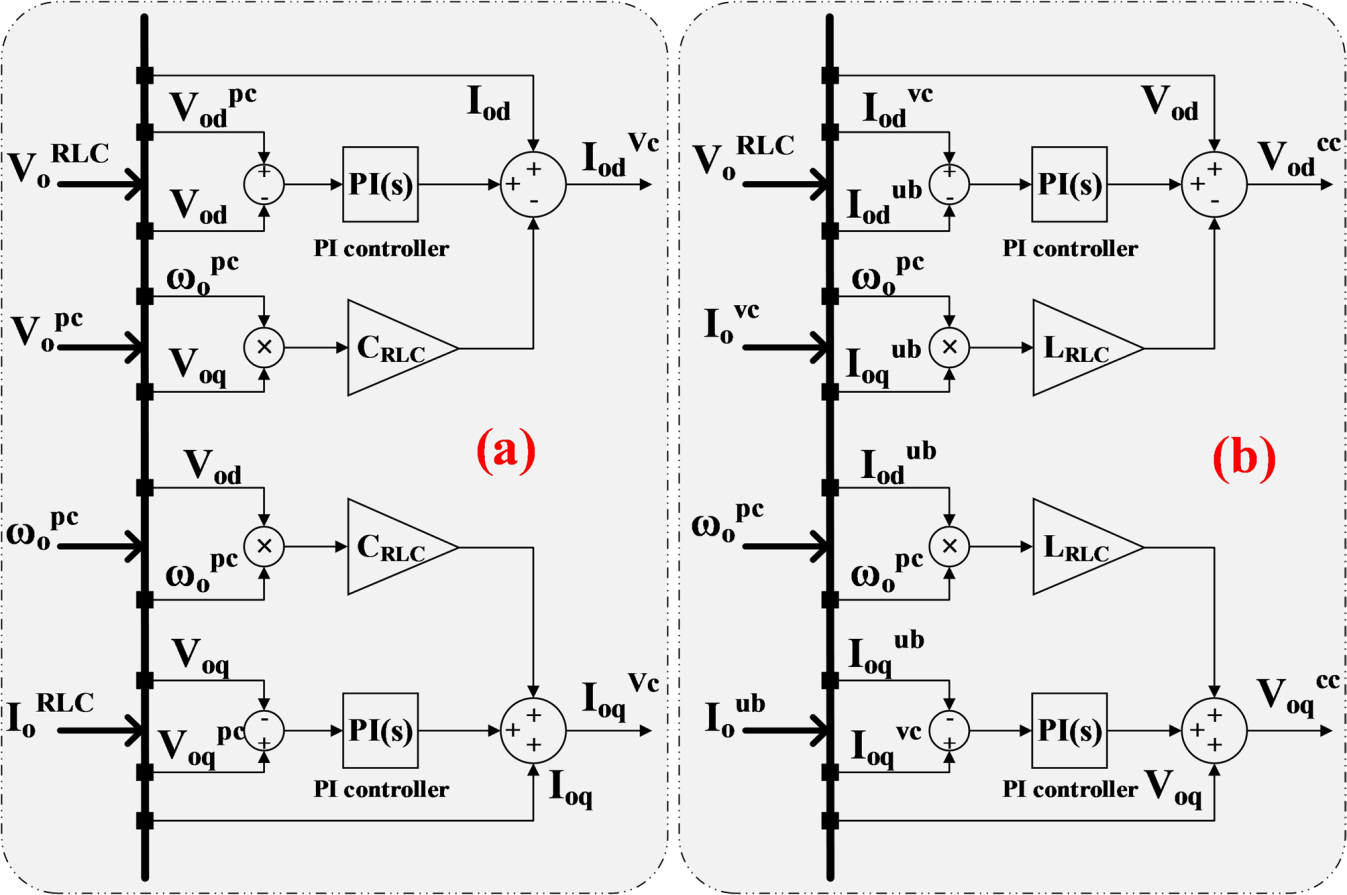
Block diagram of (**a**) voltage controller and (**b**) current controller.

Active power sharing among inverters is achieved by introducing a frequency droop characteristic into the inverter’s frequency control. Specifically, the frequency of each inverter ($\:{\omega\:}_{o}^{pc}$) is determined by subtracting the product of the droop gain ($\:{M}_{p}$) and the measured active power from the nominal frequency ($\:{\omega\:}_{r}$). Reactive power sharing, on the other hand, is implemented by applying a voltage droop in the output voltage of the power controller in the *dq*-reference frame, where $\:{V}_{r}$ represents the nominal voltage, $\:{N}_{p}$ is the droop gain for reactive power. The control strategy aligns the voltage vector with the *d*-axis of the inverter’s reference frame, while the q-axis component is maintained at zero. In a similar fashion, Fig. 4 presents the block diagrams of the voltage and current control loops, both of which are implemented using a classical proportional–integral (PI) control structure to ensure stable and accurate regulation of system variables.

### Dynamic modeling of an IMG

To accurately estimate the dynamic states in an IMG, the KF algorithm utilizes both measurable control inputs and unmeasured state variables. The control inputs consist of all quantities measurable by PMUs. The measured signals act as known external inputs to the KF. In contrast, the system states represent the unmeasured variables, such as line currents, load currents, and bus voltages that are not directly observable by PMUs.

**Fig. 5:**
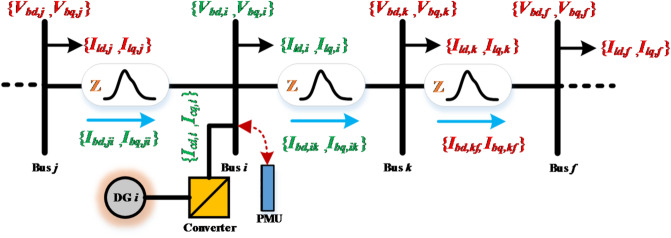
Single-line diagram of an IMG.

A single-line diagram depicting the dynamic parameters of an IMG is illustrated in Fig. 5. As shown, the PMU-installed buses provide access to key electrical quantities expressed in the *dq* reference frame, highlighted in green. These include: bus voltage $\:{V}_{bd,i}$, $\:{V}_{bq,i}$, line currents of adjacent lines $\:{I}_{bd,ji}$, $\:{I}_{bq,ji}$, $\:{I}_{bd,ik}$, $\:{I}_{bq,ik}$ (incoming and outgoing), load current $\:{I}_{ld,i}$, $\:{I}_{lq,i}$ and the output current of the converters $\:{I}_{od,i}$, $\:{I}_{oq,i}$. Together, these measured variables provide the essential input data for the KF, enabling accurate estimation of the remaining unknown system states throughout the network. These unknown variables are highlighted in red in Fig. 5.

DSE in IMGs relies on mathematical modeling of system components, typically represented by differential equations. In this context, a time-domain dynamic model is developed to represent key IMG components, including DG units, transmission lines, and loads. The dynamic behavior of all these elements is formulated using differential equations in the *dq* reference frame, based on their respective state variables. As depicted in Fig. 5, the DG unit injects output currents ($\:{I}_{od,i}$, $\:{I}_{oq,i}$) into its associated bus *i* via a VSI. The detailed dynamic model of a droop-controlled DG unit is provided in^26^. Loads within the IMG are modeled using their equivalent RL impedances, with the dynamics of the *d*-axis and *q*-axis load currents governed by Eqs. (1) and (2), respectively. Similarly, the dynamic behavior of transmission lines between buses *i* and *j* is captured by Eqs. (3) and (4)^26^.

To model bus voltages, a sufficiently large virtual resistance $\:{\mathrm{R}}_{\mathrm{n}}$ is assumed between each bus and the ground. Under this assumption, the *d*-axis and *q*-axis components of the bus voltage at bus *i* are defined in Eqs. (5) and (6). This formulation enables a unified dynamic representation of the IMG suitable for use in the KF-based DSE framework.

1 $$\:{\dot{I}}_{{ld}_{i}}\left(t\right)=\frac{1}{{L}_{{l}_{i}}}\left({V}_{bd,i}-{R}_{{l}_{i}}.{I}_{l{d}_{i}}+{\omega\:}_{c}.{L}_{{l}_{i}}.{I}_{l{q}_{i}}\:\right)\:$$

2 $$\:{\dot{I}}_{{lq}_{i}}\left(t\right)=\frac{1}{{L}_{{l}_{i}}}\left({V}_{bq,i}-{R}_{{l}_{i}}.{I}_{l{q}_{i}}-{\omega\:}_{c}.{L}_{{l}_{i}}.{I}_{l{d}_{i}}\right)$$

3 $$\:{\dot{I}}_{b{d}_{ij}}\left(t\right)=\frac{1}{{L}_{{b}_{ij}}}\left({V}_{bd,j}-{V}_{bd,i}-{R}_{{b}_{ij}}.{I}_{b{d}_{ij}}+{\omega\:}_{c}.{L}_{{b}_{ij}}.{I}_{b{q}_{ij}}\right)$$

4 $$\:{\dot{I}}_{b{q}_{ij}}\left(t\right)=\frac{1}{{L}_{{b}_{ij}}}\left({V}_{bq,j}-{V}_{bq,i}-{R}_{{b}_{ij}}.{I}_{b{q}_{ij}}-{\omega\:}_{c}.{L}_{{b}_{ij}}.{I}_{b{d}_{ij}}\right)$$

5 $$\:{V}_{bd,i}={R}_{n}\left[{I}_{o{d}_{i}}-{I}_{ld,i}+\sum\:_{\:}^{\:}{I}_{{bd}_{i\left(in\right)}}-\sum\:_{\:}^{\:}{I}_{{bd}_{i\left(out\right)}}\right]$$

6 $$\:{V}_{bq,i}={R}_{n}\left[{I}_{o{q}_{i}}-{I}_{lq,i}+\sum\:_{\:}^{\:}{I}_{{bq}_{i\left(in\right)}}-\sum\:_{\:}^{\:}{I}_{{bq}_{i\left(out\right)}}\right]$$

In Eqs. (1, 2, 3, 4), $\:\dot{I}$ denotes the time derivative of the current *I*. By utilizing the measured information together with the set of Eqs. (1, 2, 3, 4, 5, 6), the system states can be effectively estimated using a KF algorithm. It is noteworthy that in Eqs. (1, 2, 3, 4, 5, 6), the absence of multiplication between current and voltage variables ensures that the mathematical model remains linear. The use of such linear equations within the KF, an algorithm fundamentally designed for linear systems, enables effective estimation of the dynamic states in a linear state-space representation. This inherent linearity in both the mathematical model and the filtering algorithm ensures consistency, which improves the accuracy and robustness of the DSE process.

Although the IMG exhibits inherent nonlinearity in its native *abc* frame, the adopted dynamic model formulation is conducted entirely within the *dq* reference frame, rendering the governing equations fully linear. Consequently, all variations in the operating point are naturally captured through the time-varying state measurements provided by the PMUs entering the estimator as inputs. Given that the proposed framework already integrates a computationally intensive neural network-based GAIN module, maintaining a lightweight estimation core is paramount. Adopting nonlinear estimators, such as the EKF with its $\:O\left({n}^{3}\right)$ + Jacobian computation, the UKF at $\:{O\left(2n+1\right)}^{3}$, or PFs at $\:O\left({N}_{p}n\right)$, would substantially escalate the total computational burden without offering accuracy improvements for an already linearized dq -domain model. Therefore, the linear KF remains the most computationally efficient and theoretically consistent choice for DSE in this IMG architecture.

### Probabilistic line impedance

As illustrated in Fig. 5, the line impedance in the IMG is modeled from a probabilistic perspective to better capture real-world operational uncertainties. In particular, the line resistance $\:{R}_{b}$, which varies with ambient temperature, is treated as a random variable. According to Eq. (7), this resistance is defined as a temperature-dependent function, and its stochastic nature is represented using probability distributions. To further quantify the impact of temperature fluctuations on DSE, a normal distribution, as described in Eq. (8), is employed. Assuming that ambient temperature follows a normal distribution, the corresponding line resistance $\:{R}_{b}$ is also modeled as a normally distributed variable. By incorporating this probabilistic resistance into the dynamic system equations (Eqs. (3) and (4)), stochastic behavior is introduced into the model. To systematically evaluate the influence of this uncertainty on estimation performance, a series of MCSs is performed.

7 $$\:{R}_{b}={R}_{o}(1+\alpha\:\:\varDelta\:T)$$

8 $$\:T=\:\mu\:\:+\:\sigma\:\:\mathcal{r}$$

In Eqs. (7) and (8), $\:{R}_{o}$ is the resistance at the reference temperature, $\:\alpha\:$ is the temperature coefficient of specific resistance, and $\:\varDelta\:T$ shows the temperature changes. Additionally, is the average ambient temperature, shows how much the temperature varies, and $\:\mathcal{r}$ is a random variable that follows a normal distribution with a mean of 0 and a standard deviation of 1.

## Proposed methodology

This paper proposes a hybrid approach for DSE in IMGs by integrating the KF with a deep learning-based GAIN. The KF is employed for its simplicity, linearity, and low computational cost, making it suitable for real-time applications. Although the IMG is nonlinear, fast PMU updates anchor each estimation step to a recent operating point where a measurement-driven small-signal description is adequate; with sufficient PMU coverage and update rate, the KF converges reliably and residual nonlinearities behave as bounded process uncertainty, so tracking accuracy is maintained even during dynamic events. For larger excursions, the operating point is refreshed (re-linearized), preserving fidelity to the nonlinear behavior while keeping real-time efficiency.

For the linearized IMG model considered in this work, the KF was chosen as the core estimator because it represents the theoretically unique linear minimum mean square error (LMMSE) estimator under Gaussian noise conditions^48^, offering superior guarantees compared to other linear techniques such as least square (LS) and WLS, which ignore system dynamics and involve cubic complexity, or H∞ filtering, which yields more conservative estimates^49^. Recursive least square (RLS) reduces computational cost but does not incorporate noise covariance matrices and therefore cannot achieve LMMSE optimality^50,51^. Addressing the concerns regarding initial state dependency and convergence under faults, it is noted that for observable linear systems, the KF is asymptotically stable, ensuring that the influence of the initial state diminishes over time as the Riccati recursion converges to a steady state^48^.

Furthermore, to counteract performance degradation caused by faulty or missing measurements—a known issue since the KF relies on statistical consistency—a GAIN is integrated as a preprocessing module. GAIN processes the raw measurement vector to produce a point-wise imputed vector free of missing entries, ensuring the KF receives a complete and statistically coherent input at every time step. This architecture preserves the KF’s proven theoretical optimality while significantly enhancing its resilience to measurement anomalies and initialization uncertainty. To explicitly justify the selection of KF over alternatives, a summary of the optimality criteria and computational characteristics of these alternative estimation methods is provided in Table 3.

**Table 3:** Linear estimation methods for DSE.

Method	Uses Dynamics	Optimality Criterion	Computational Complexity	Strengths	Limitations	Ref.
LS/WLS	No	Static least-squares	$\:O\left({m}^{3}\right)$	Simple; widely used	Ignores dynamics; not real-time	^50^
RLS	Limited	No LMMSE guarantee	$\:O\left({n}^{2}\right)$	Fast; recursive	Poor noise modeling	^50,51^
H∞ Filter	Yes	Worst-case (min–max)	$\:O\left({n}^{2}\right)$	Robust to uncertainty	Conservative; requires tuning; not LMMSE	^49^
KF	Yes	LMMSE optimal	$\:O\left({n}^{2}\right)$	Mainstream DSE; optimal accuracy	Requires Q, R tuning	^48,49^

To address the challenge of missing or incomplete measurement data, the GAIN model is incorporated, leveraging its hint mechanism to generate realistic imputations and outperform conventional GAN-based methods. By combining the strengths of both techniques, KF’s efficiency in linear estimation and GAIN’s robustness in data imputation, the proposed framework enhances the accuracy and reliability of DSE under practical conditions involving data loss. Detailed descriptions of each method are provided in the following sections.

### Kalman filter

**Fig. 6:**
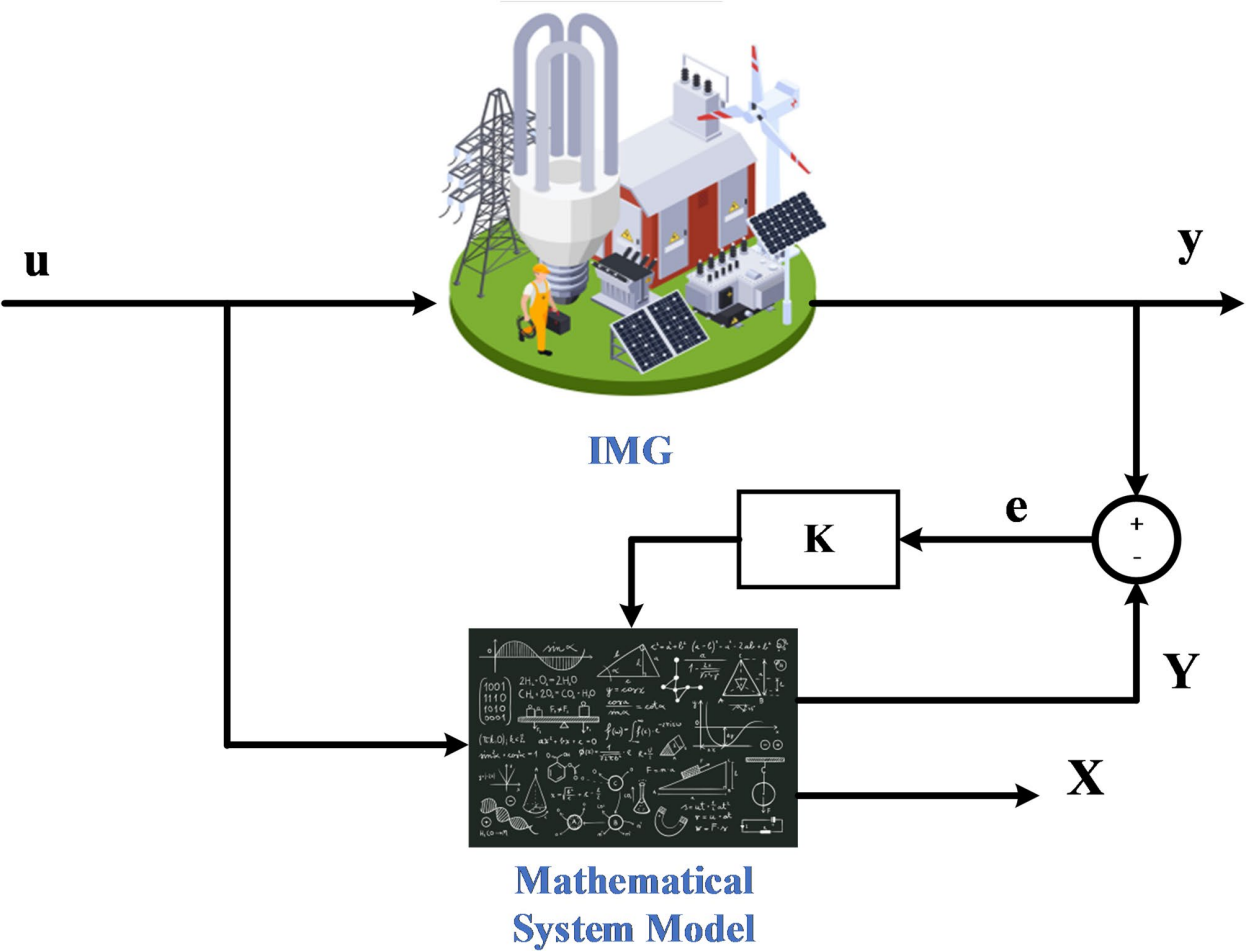
Block diagram of KF algorithm.

The KF is a widely adopted recursive algorithm used to estimate the states and parameters of systems under conditions of noise and uncertainty. Owing to its model-based structure and computational efficiency, the KF is especially well-suited for real-time monitoring and control applications. In the context of IMGs, where accurate knowledge of internal states, such as voltages, currents, and power flows, is critical for reliable operation, the KF offers a robust framework for DSE. By integrating system dynamics with real-time measurements, the KF continuously refines its estimates, enabling precise tracking of the system’s behavior even in the presence of measurement noise and system disturbances. A generalized schematic of the KF operation is shown in Fig. 6.

The KF operates through two sequential phases: prediction and update. In the prediction phase, the system’s next state is estimated based on its current state and control inputs, as modeled by the state transition equation (Eq. (9)). Simultaneously, the associated uncertainty is projected forward using Eq. (11), which accounts for process noise. In the update phase, this prediction is refined using new measurement data via the measurement equation (Eq. (10)). The Kalman gain (Eq. (12)) determines the optimal weighting between the model prediction and the actual observation, based on their respective error covariances. The state estimate is then corrected using Eq. (13), which incorporates the discrepancy between predicted and observed values, and the error covariance is updated accordingly using Eq. (14). Through this recursive process, the KF generates progressively accurate state estimates, even in the presence of noise and partial data loss. This makes it particularly valuable for DSE in IMGs, where reliable real-time monitoring is essential for maintaining stability and performance.

9 $$\:\stackrel{-}{X}\left(t+1\right)=A\:X\left(t\right)+B\:u\left(t\right)+w\left(t\right)$$

10 $$\:Y\left(t+1\right)=C\:X\left(t+1\right)+D\:u\left(t+1\right)+v\left(t+1\right)$$

11 $$\:\stackrel{-}{\mathcal{P}}\left(t+1\right)=A\:\mathcal{P}\left(t\right)\:{A}^{T}+\mathcal{Q}$$

12 $$\:K\left(t+1\right)=\stackrel{-}{\mathcal{P}}\left(t+1\right)\:{C}^{T}\:{\left(C\:\stackrel{-}{\mathcal{P}}\left(t+1\right)\:{C}^{T}+\mathcal{R}\right)}^{-1}$$

13 $$\:X\left(t+1\right)=\stackrel{-}{X}\left(t+1\right)+\:K\left(t+1\right)\:\left(Y\left(t+1\right)-C\:\stackrel{-}{X}\left(t+1\right)-D\:u\left(t+1\right)\right)$$

14 $$\:\mathcal{P}\left(t+1\right)=\left(\mathbb{I}-K\left(t+1\right)\:C\right)\:\stackrel{-}{\mathcal{P}}\left(t+1\right)$$

In Eqs. (9)-(14), $\:X\left(t\right)$ is the state vector, $\:u\left(t\right)$ is the control input, $\:A$ and $\:B$ are system matrices, and $\:w\left(t\right)\sim\:\mathcal{N}(0,\mathcal{Q})$ represents the process noise. Also, $\:Y\left(t\right)$ is the observation vector, $\:C$ and $\:D$ are measurement matrices, and $\:v\left(t\right)\sim\:\mathcal{N}(0,\:\mathcal{R})$ is measurement noise. Furthermore, the matrices $\:\mathcal{P}$, $\:\mathcal{Q}$, $\:K$, and $\:\mathcal{R}$ correspond to the state estimate covariance, process noise covariance, Kalman gain, and measurement noise covariance matrices, respectively. Finally, throughout Eqs. (9, 10, 11, 12, 13, 14), the overbar (“¯”) on $\:X$ and $\:\mathcal{P}$ denotes a priori (predicted) quantities.

The KF algorithm requires as inputs the matrices *A*, *B*, *C*, *D*, $\:\mathcal{Q}$, and $\:\mathcal{R}$, along with the initial SE, the initial state covariance matrix, and the control input *u*. Its outputs are two arrays: one containing the estimated state vectors and the other containing the corresponding error covariance matrices. The algorithm operates iteratively over each time step *t*, from 1 to the total number of samples. In each iteration, the process begins with the prediction phase, where the next state and its associated error covariance are estimated using Eqs. (9) and (11). This is followed by the Kalman gain computation via Eq. (12), which determines how the prediction should be adjusted based on new measurements. In the subsequent update phase, the SE and its covariance are refined using Eqs. (13) and (14), incorporating the current measurement. After each iteration, the updated estimates and covariances are stored. This process continues until all samples are processed. Finally, the algorithm returns the complete set of estimated states and their corresponding covariance matrices.

As described in Sect. 2.2, the control inputs to the KF include all quantities measured by PMUs—such as converter output currents, load and line currents, and bus voltages—which are highlighted in green in Fig. 5. In contrast, the state vector consists of all unknown voltage and current variables within the system, shown in red in Fig. 5.

### GAIN for missing data imputation

In IMGs, missing measurement data causes significant challenges and inaccuracies in DSE. These issues arise from factors such as equipment failure, sudden power outages due to distribution network problems or other causes, electromagnetic disturbances, software malfunctions, and failures in the measurement system, including devices and connections, resulting in missing data. To enhance the robustness of the proposed model-based approach, this paper employs a GAIN structure^52^. The GAIN is designed to estimate missing data by leveraging the adversarial training paradigm. It consists of two main components: a generator ($\:\mathbb{G}$) and a discriminator ($\:\mathbb{D}$). The generator and discriminator are modeled using a multilayer neural network, both having a structure similar to Table 4, and the training mechanism for this network utilizes the backpropagation method.

**Table 4:** Structure used for the generator and discriminator in GAIN networks.

Layer	Dimensions	Weights	Biases	Parameters
Input	[B,96]	-	-	0
Layer 1	[B,48]	[96,48]	48	4656
Layer 2	[B,96]	[48,96]	96	4704
Layer 3	[B,144]	[96,144]	144	13,968
Layer 4	[B,144]	[144,144]	144	20,880
Layer 5	[B,96]	[144,96]	96	13,920
Layer 6	[B,48]	[96,48]	48	4656
Layer 7	[B,48]	[48,48]	48	2352

**Fig. 7:**
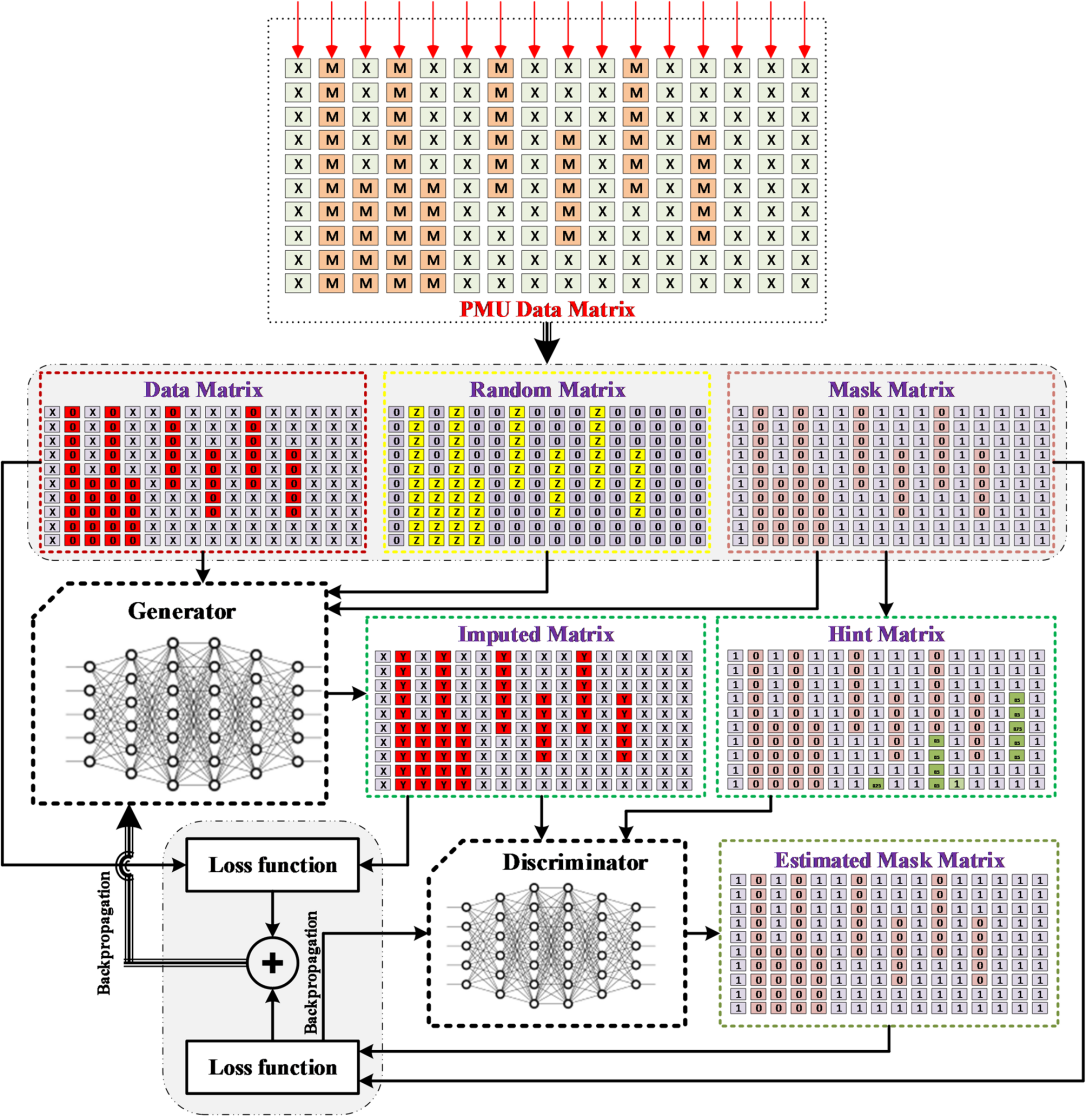
GAIN network.

The overall architecture of the deep network is shown in Fig. 7^53^, which illustrates the process of compensating for missing data in the DSE of IMGs using the GAIN framework. In this figure, the entries of “M” indicate the presence of missing data, while “X” contains the available measurements. It is assumed that missingness occurs at the PMU level—i.e., when a value in “M” indicates missing data, it corresponds to the loss of all measurements from the associated PMU at that time step. Initially, missing entries in the data matrix, including measurements from PMUs, are filled with random values. These preliminary imputations serve as inputs to a neural network trained to iteratively refine and accurately estimate the missing values. Auxiliary matrices, including the mask that indicates missing data locations and the hint matrix that provides partial guidance, assist the GAIN model in effectively identifying and imputing the missing states. This approach enables robust reconstruction of incomplete dynamic measurements. Consequently, it improves the reliability and accuracy of IMG state estimation.

In conventional GANs, the generator creates realistic data from random noise, while the discriminator learns to distinguish between real and fake samples. However, in data imputation tasks, the objective shifts from generating entirely new data to filling in missing values within existing datasets. Without explicit guidance, the discriminator in a GAN-based imputation framework struggles to identify which input values are genuinely observed and which are imputed, often leading to unstable or ineffective training. To overcome this, GAIN introduces a hint mechanism that provides the discriminator with partial knowledge about observed components of the input data. This hint vector is a partially randomized binary mask that reveals parts of the mask vector, indicating observed (1) and missing (0) data points. By receiving both the imputed data and the hint vector, the discriminator’s role is to predict the mask vector—that is, to distinguish real values from imputed ones. This setup forces the generator to improve the quality of imputed values to the point where the discriminator can no longer reliably differentiate between observed and imputed data. In essence, GAIN’s key innovation, the hint mechanism, guides the adversarial process by supplying the discriminator with partial information on observed data locations. This targeted guidance significantly enhances the realism of imputations, making GAIN a highly effective and robust method for data imputation in scenarios with missing values.

In GAIN, the generator aims to impute missing data using three inputs: the observed data matrix $\:{X}_{k}$, a mask matrix $\:\mathcal{M}$, and a noise vector $\:\mathcal{Z}$. The generator combines these inputs to estimate the missing values. Notably, the noise only affects the missing components, as defined in Eq. (15)^52^.

15 $$\:\stackrel{-}{\mathcal{Z}}=\left(1-\mathcal{M}\right)\odot \mathcal{Z}$$

In (15), $\:\mathcal{M}$ is a binary mask matrix indicating the positions of missing data, while $\:\mathcal{Z}$ is a noise matrix generated by a predefined noise function. The symbol $\odot$ represents element-wise multiplication. Based on these definitions, the overall data space can be expressed as the union of two subspaces: the observed data $\:{X}_{k}$ and the missing (to be imputed) data $\:{\stackrel{\sim}{X}}_{k}$, as follows:

16 $$\:{\widehat{X}}_{k}=\left\{\begin{array}{c}{X}_{k}\:\:\:\:\:\:\:\:\:\:\:\:\:\:\:\:\:\:\:{\mathcal{M}}_{k}=1\\\:{\stackrel{\sim}{X}}_{k}\:\:\:\:\:\:\:\:\:\:\:\:\:\:\:\:\:\:\:{\mathcal{M}}_{k}=0\end{array}\right.$$

In this context, when $\:{\mathcal{M}}_{k}=1$, the corresponding value in $\:{X}_{k}$ represents the observed (available) data. Conversely, when $\:{\mathcal{M}}_{k}=0$, the value is considered missing and is replaced by $\:{\stackrel{\sim}{X}}_{k}$, which is generated using the noise input.

The discriminator aims to distinguish real data from synthetic data generated by the generator, thereby guiding the generator toward learning the true data distribution. Denoted by $\:\mathbb{G}$, the generator feeds synthetic data into the discriminator, whose input can be expressed as Eq. (17), where $\:{\widehat{X}}_{k}$ and $\:\stackrel{-}{{X}_{k}}$ are the data matrix and the imputed matrix, respectively. The discriminator, like the generator, is implemented as a multilayer neural network.

17 $$\:\stackrel{-}{{X}_{k}}=\mathcal{M}\odot {X}_{k}+(1-\mathcal{M})\odot\:\mathbb{G}\left({\widehat{X}}_{k},\mathcal{M},\stackrel{-}{\mathcal{Z}}\right)$$

Since the generator is capable of producing data across various distributions, it is critical to inform the discriminator about the locations of the missing data. To facilitate this, a Hint matrix is introduced. This matrix serves as a stochastic variable defined over the same space as the input data and partially reveals information about the missingness pattern to the discriminator. It is formally defined as Eq. (18), where $\:\mathcal{B}$ is a binary matrix sampled from a Bernoulli distribution, controlling the level of hint provided.

18 $$\:H=\mathcal{B}\odot\mathcal{M}+0.5\times\:(1-\mathcal{B})$$

The primary objective of GAIN is to maximize the probability of correctly distinguishing between original and imputed data, as formulated in Eq. (19). To achieve this goal, the GAIN architecture enhances the discriminator’s performance by providing it with additional information in the form of hint vectors (*H*). On the other hand, the generator is trained to minimize the likelihood that the discriminator can identify the generated data as fake, as expressed in Eq. (20). Furthermore, to preserve the integrity of observed data, a mean squared error (MSE) term is incorporated into the generator’s objective function, as shown in Eq. (21). This ensures that imputation affects only the missing entries, maintaining the accuracy of known data.

19 $$\:\underset{\mathbb{D}}{\mathbf{max}}\mathrm{U}\left(\mathbb{D},\mathbb{G}\right)={\mathbb{E}}_{\stackrel{-}{{X}_{k}},\mathcal{M},\mathcal{Z},H}[{\mathcal{M}}^{T}.\mathrm{log}\mathbb{D}\left(\stackrel{-}{{X}_{k}},H\right)+{\mathbb{E}}_{\stackrel{-}{{X}_{k}},\mathcal{M},\mathcal{Z},H}[{\left(1-\mathcal{M}\right)}^{T}.\:\:\mathrm{log}(1-\mathbb{D}\left(\stackrel{-}{{X}_{k}},H\right))]$$

20 $$\:\underset{\mathbb{G}}{\mathbf{min}}\mathrm{U}\left(\mathbb{D},\mathbb{G}\right)={\mathbb{E}}_{\stackrel{-}{{X}_{k}},\mathcal{M},\mathcal{Z},H}[{\left(1-\mathcal{M}\right)}^{T}\mathrm{*}\mathrm{log}(1-\mathbb{D}\left(\stackrel{-}{{X}_{k}},H\right))]$$

21 $$\:\underset{\mathbb{G}}{\mathbf{min}}\mathrm{M}\mathrm{S}\mathrm{E}({\widehat{X}}_{k},\stackrel{-}{{X}_{k}},\mathcal{M})=\sum\:\mathcal{M}\odot\left[{\left({\widehat{X}}_{k}-\stackrel{-}{{X}_{k}}\right)}^{2}\right]$$

The adaptive moment estimation (Adam) optimizer^54^ is one of the most widely used methods for tuning the parameters of data-driven models due to its efficiency and reliability. Adam is an advanced variant of stochastic gradient descent that adapts the learning rate by estimating the first and second moments of the gradients. This adaptation enables faster convergence and more stable training of neural networks over iterative epochs. In this study, Adam is employed to optimize the model’s hyperparameters, helping to prevent both overfitting and underfitting, thereby enhancing the model’s generalization capability.

AE and VAE are used as baselines to evaluate the performance of the proposed GAIN model. Hyperparameters for GAIN, AE, and VAE were selected via grid search on a held-out validation set; each model was tuned under the same training protocol, and the best configuration was chosen by validation loss/NRMSE averaged over random PMU-dropout masks. Table 5 summarizes the selected GAIN settings: learning rate 0.005 with Adam, a maximum of 10,000 epochs, mini-batch size 512, reconstruction-loss weight 0.8, ReLU activations in hidden layers, Sigmoid at the output, Xavier initialization, and Adam for both generator and discriminator.

Table 6 summarizes the key distinctions between AE and VAE in terms of model architecture, training strategy, and loss functions. AE is a deterministic model that compresses input data into a latent space and reconstructs it directly. In contrast, VAE is a probabilistic generative model that learns a distribution over the latent space and employs the reparameterization trick to enable efficient gradient-based optimization. While AE minimizes only the reconstruction error (e.g., MSE), VAE incorporates an additional regularization term, the Kullback–Leibler divergence, to ensure that the learned latent distribution approximates a standard normal distribution. Both models were implemented using PyTorch, trained with the Adam optimizer at a learning rate of 0.001 over 1000 epochs. The performance of each model was evaluated using the MSE metric. As reported in Table 6, the VAE offers a significant advantage in modeling uncertainty and generating more diverse and robust data compared to the standard AE.

**Table 5:** Hyperparameters used in the proposed GAIN model.

Hyperparameter	Value	Description
Learning rate	0.005	Step size for Adam optimizer
Number of epochs (iterations)	10,000 (max)	Maximum number of training iterations
Mini-batch size	512	Number of samples per training batch
Reconstruction loss weight	0.8	Balancing factor between adversarial and reconstruction losses
Optimizer	Adam	Optimization algorithm for both generator and discriminator
Activation function	ReLU/Sigmoid	ReLU for hidden layers, Sigmoid for output layers
Initialization	Xavier	Weight initialization method

**Table 6:** Summary of AE and VAE architectures and training configuration.

Feature	AE	VAE
Model type	Deterministic autoencoder	Probabilistic latent variable model
Encoder architecture	Input → (2×input_dim) → ReLU → input_dim	Input → (2×input_dim) → ReLU → [µ, logσ²]
Latent sampling	Not applicable	Reparameterization trick: z = µ + σ ⊙ ε, where ε ~ N(0, 1)
Decoder architecture	input_dim → (2×input_dim) → ReLU → input_dim	Latent_dim → (2×input_dim) → ReLU → input_dim
Loss function	MSE	MSE + Kullback-Leibler divergence
Optimizer	Adam	Adam
Learning rate	0.001	0.001
Epochs	1000	1000
Regularization	None	KL Divergence
Implementation framework	PyTorch	PyTorch
Evaluation metric	RMSE	RMSE

**Fig. 8:**
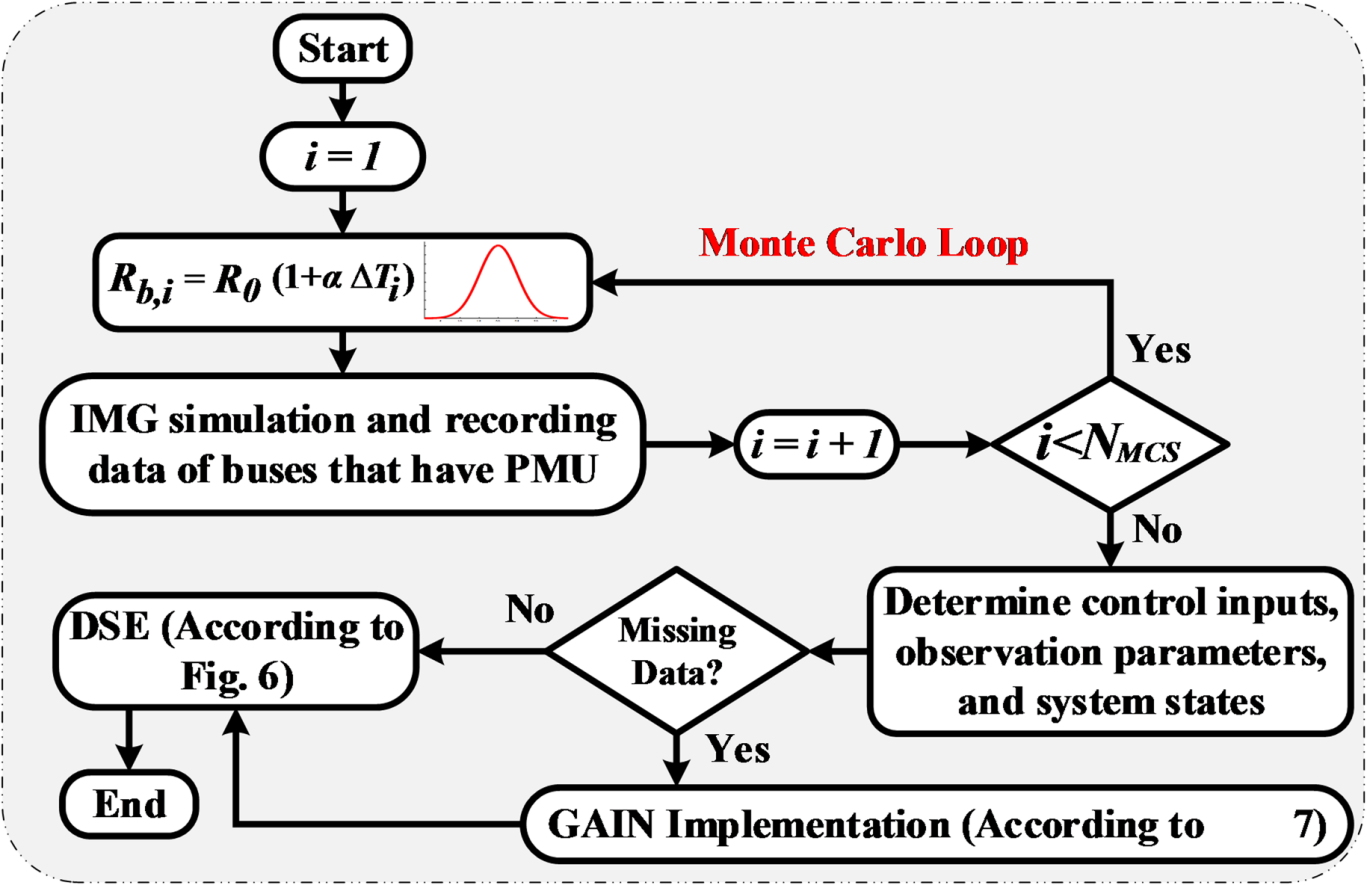
Overall flowchart of the proposed framework.

The overall flowchart of the proposed framework for DSE of IMGs, considering uncertainties, is illustrated in Fig. 8. The framework consists of four main components: (i) MCS uncertainty modeling, (ii) IMG implementation, (iii) deep learning (GAIN), and (iv) network-based DSE (KF). The process begins with initialization of the IMG simulation and PMU data acquisition. Measurement data are collected from buses equipped with PMUs. The dataset is analyzed; if missing measurements are detected, GAIN estimates and imputes them. Using the completed dataset, control inputs, observation variables, and system states are defined, and the KF is then applied to perform DSE. The estimation results are finally prepared for evaluation or further analysis.

## Simulation results and discussion

**Fig. 9:**
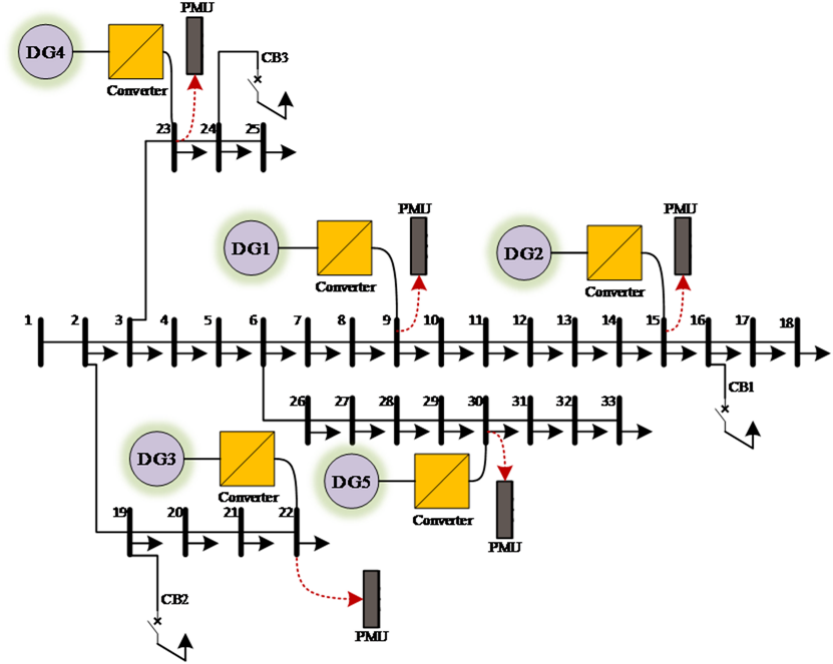
Islanded distribution feeder of IEEE 33 bus.

This section evaluates the proposed framework on the islanded IEEE 33-bus distribution feeder with five DG units and PMU measurements distributed across the network, as shown in Fig. 9^26^. The hybrid KF–GAIN approach scales to larger systems without changing the method. This framework is inherently scalable because its components operate based on localized, real-time statistical relationships derived from PMU measurements rather than relying on global network topology dependencies. The GAIN module’s performance is enhanced, not hindered, by larger systems as increased PMU count improves statistical redundancy for imputation. Furthermore, the KF operates on the modular *dq*-domain model, ensuring that the estimation logic remains consistent regardless of system size. The selection of the smaller test microgrid was purely methodological, aimed at achieving a transparent and controlled evaluation of framework performance under varied loss scenarios, confirming applicability to larger, more complex IMGs without conceptual modification.

In the proposed hybrid KF–GAIN approach, at each time step, GAIN fills any missing PMU readings and the linear KF estimates the state from the completed data; as systems grow, adequate PMU placement maintains observability and filter convergence. The pipeline uses standard time-stamped PMU data and meets real-time requirements, enabling drop-in integration with existing IMG monitoring infrastructure without hardware changes.

The PMUs measure the parameters related to the output current from the converter units, bus voltages, line currents, and the current of loads connected to the bus in the *dq*-frame. The test case consists of five VSIs, each with a capacity of 1 MW. The system operates at a nominal frequency of 60 Hz and a nominal voltage of 12,660 V, while PMUs sample data at a rate of 256 samples per cycle^55^. Although the PMUs acquire measurements internally at 256 samples per cycle, the DSE framework processes data at the PMU reporting rate of 30 samples per second, which reflects the actual rate at which phasor measurements are delivered for real-time DSE. All reported estimation errors and computational times correspond to this effective reporting rate. All simulations were performed on a workstation equipped with an Intel Core i7 CPU, 16 GB RAM, and SSD storage. The parameter values necessary for the network simulation are provided in Table 7.

**Table 7:** Parameter setting for VSI simulation.

Parameter	$\:{\boldsymbol{R}}_{\boldsymbol{R}\boldsymbol{L}\boldsymbol{C}}$	$\:{\boldsymbol{L}}_{\boldsymbol{R}\boldsymbol{L}\boldsymbol{C}}$	$\:{\boldsymbol{C}}_{\boldsymbol{R}\boldsymbol{L}\boldsymbol{C}}$	$\:{\boldsymbol{R}}_{\boldsymbol{R}\boldsymbol{L}}$	$\:{\boldsymbol{L}}_{\boldsymbol{R}\boldsymbol{L}}$	$\:{\boldsymbol{K}}_{{\boldsymbol{P}}_{\boldsymbol{v}\boldsymbol{c}}}$	$\:{\boldsymbol{K}}_{{\boldsymbol{I}}_{\boldsymbol{v}\boldsymbol{c}}}$	$\:{\boldsymbol{K}}_{{\boldsymbol{P}}_{\boldsymbol{c}\boldsymbol{c}}}$	$\:{\boldsymbol{K}}_{{\boldsymbol{I}}_{\boldsymbol{c}\boldsymbol{c}}}$
Value	0.01	5e-05	5e-05	0.023	3.5e-05	0.286	5.95e + 02	54.97	1.57e + 03

In this study, dynamic loads—activated or deactivated during the multi-second simulation—along with DG units are strategically located on the lateral branches of the feeder, particularly in the downstream and weakly connected sections of the IEEE 33-bus IMG. These branches exhibit high voltage sensitivity, low electrical stiffness, and strong radial power-flow coupling. In addition, the network is characterized by distinct operating regions, including the main feeder backbone (buses 1–18), downstream laterals (buses 19–33), and a local lateral area (buses 23–25), which together define different observability conditions. Accordingly, PMU loss scenarios are considered in these weak and highly sensitive regions to establish a worst-case evaluation environment for the proposed KF–GAIN estimator.

In the absence of an upstream grid reference, the converter-interfaced DG units (DG1–DG5) jointly regulate voltage and frequency in islanded operation. The main feeder backbone (buses 1–18), where DG1 and DG2 are connected, serves as the primary power support path, where disturbances propagate rapidly under PMU loss conditions. In contrast, downstream laterals (buses 19–33), hosting DG3, DG5, and dynamic loads, experience larger voltage variations and stronger radial coupling, increasing measurement redundancy that benefits the GAIN-based reconstruction. The upper lateral region (buses 23–25), associated with DG4, forms a locally stiff area with strong phasor correlation, further enhancing estimation accuracy. Overall, by enforcing PMU loss in the most weakly stiff and voltage-sensitive regions, the proposed framework is evaluated under genuinely worst-case operating conditions.

In this paper, two cases are formulated to present and evaluate the proposed approach:

*Case I:* DSE with probabilistic line impedances modeled via MCS. 

* Case II:* DSE with missing data imputed using the GAIN model to restore observability and enhance accuracy.

### Case I

This section provides a comprehensive analysis of the DSE simulation using the KF. In this manner, the uncertainty arising from variations in line impedance will be examined to enhance the accuracy of DSEs in IMGs. For this reason, the load on bus-16 is altered by connecting an additional load of 0.6 MW and 0.2 MVAr to the grid at $\:t=2$ seconds, which is then disconnected at t = 3 s. It is assumed that no data is lost in this case, and KF is used to estimate the states.

Two error metrics, MSE and mean absolute percentage error (MAPE), have been calculated to facilitate the comparison of the DSE results. The methods for calculating MSE and MAPE are outlined in Eqs. (22) and (23), respectively.

22 $$\:MSE=\frac{1}{n}\left({\sum\:}_{i=1}^{n}{\left({y}_{{act}_{i}}-{y}_{{est}_{i}}\right)}^{2}\right)\times\:100$$

23 $$\:MAPE=\frac{1}{n}{\sum\:}_{i=1}^{n}\left(\left|\frac{{y}_{{act}_{i}}-{y}_{{est}_{i}}}{{y}_{{act}_{i}}}\right|\right)\times\:100$$

where, $\:{y}_{{act}_{i}}$, $\:{y}_{{est}_{i}}$, and $\:n$ represent the actual value of the *i*th sample obtained from simulation, the estimated value of the *i*th sample, and the total number of samples, respectively.

**Fig. 10:**
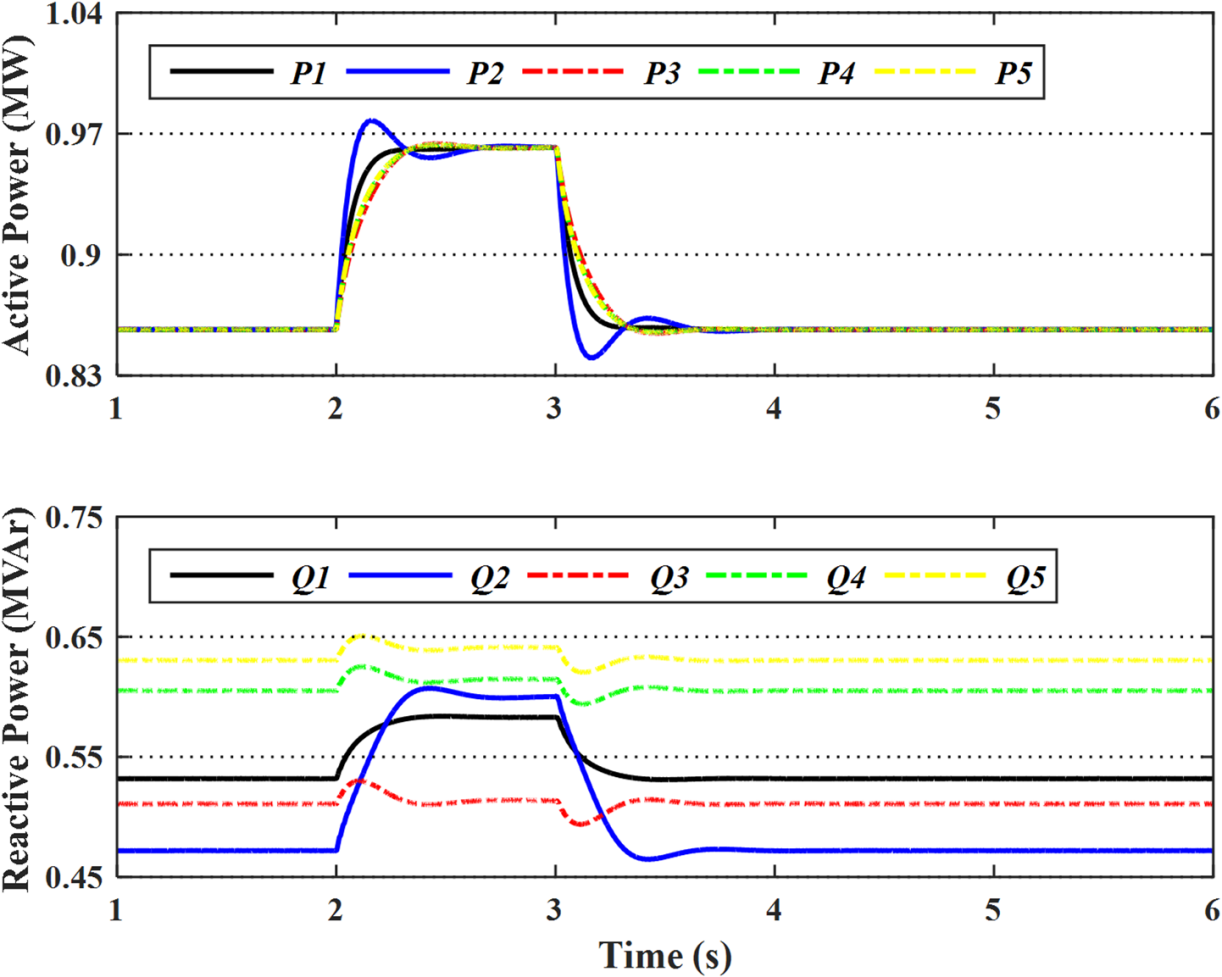
Active and reactive power generation of DGs in Case *I*.

**Fig. 11:**
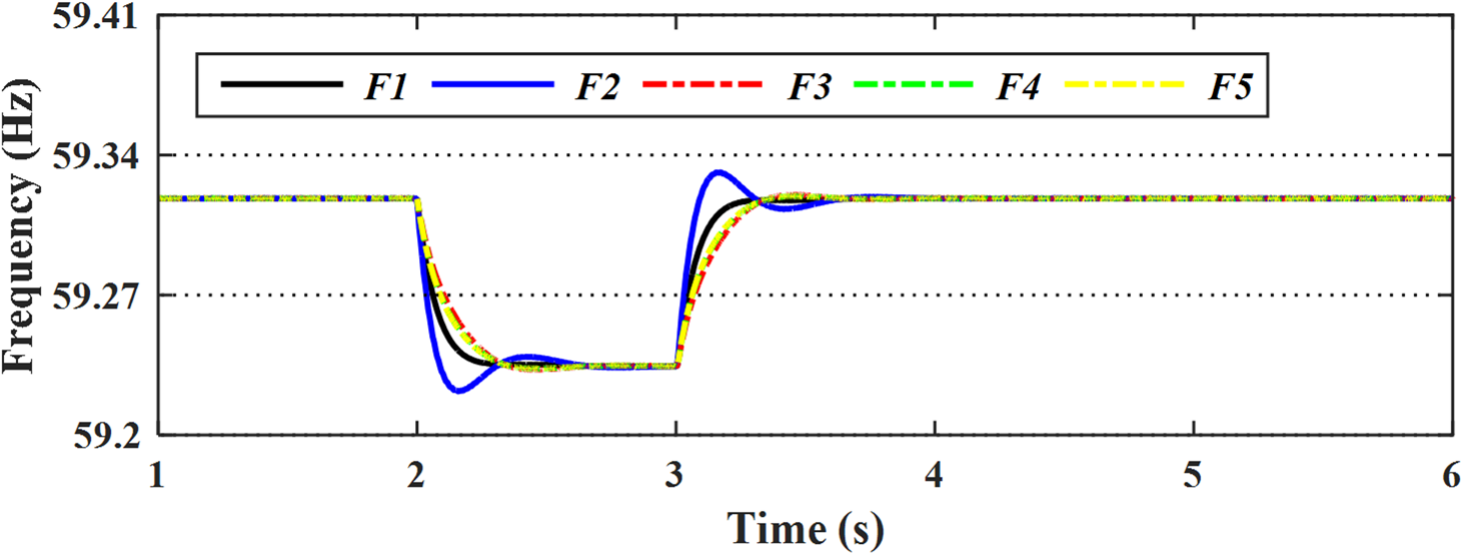
Frequency of DGs in Case *I*.

The simulation results for the active/reactive power and frequency of DGs with the same droop coefficients, as a result of the applied changes, are presented in Figs. 10 and 11, respectively. These results demonstrate that the DGs effectively tracked dynamic load variations, maintaining power balance and frequency stability throughout the simulation. The droop control mechanism successfully distributed the load among the DGs, ensuring coordinated sharing of both active and reactive power under decentralized control. Frequency deviations remained minimal, reflecting well-tuned droop characteristics that stabilize system dynamics without centralized control or communication. This strategy enables the IMG to autonomously adapt to changing conditions, enhancing resilience and operational robustness.

In total, 48 parameters are measured by the PMUs, including 18 branch currents, 10 bus voltages, 10 load currents, and 10 converter currents. These measurements correspond to five buses equipped with PMUs, DG units, and converters, as shown in Fig. 9. Specifically, for each of the five buses, the corresponding load current, converter current, and bus voltage are measured. In the *abc* reference frame, this accounts for 15 parameters, which double to 30 components in the *dq* reference frame (15 × 2). Additionally, branch currents before and after the buses with PMUs are measured; there are 9 such line currents instead of the expected 10, due to PMU number 3 being located at bus 22, the end of the feeder. Since all currents and voltages are represented in the *dq* reference frame, the number of components doubles, resulting in 18 branch current components. The absence of one line current measurement reduces the total from 20 to 18 components (10 lines × 2 components each). This measurement setup ensures comprehensive monitoring of the IMG’s electrical states.

Table 8 provides numerical values of the two evaluation indices, MSE and MAPE, to assess the performance of DSE at the four terminal buses of the studied network—buses 1, 18, 25, and 33—related to the implementation of changes in the examined feeder. The results indicate that, although bus 25 has acceptable values, it performs the worst among the buses examined due to having the highest load value throughout the feeder.

**Table 8:** Results of DSE in Case *I*.

Bus	MSE (%)	MAPE (%)
1	8.53e-4	0.0705
18	27e-4	0.2144
25	68e-4	0.7176
33	26e-4	0.2660

**Fig. 12:**
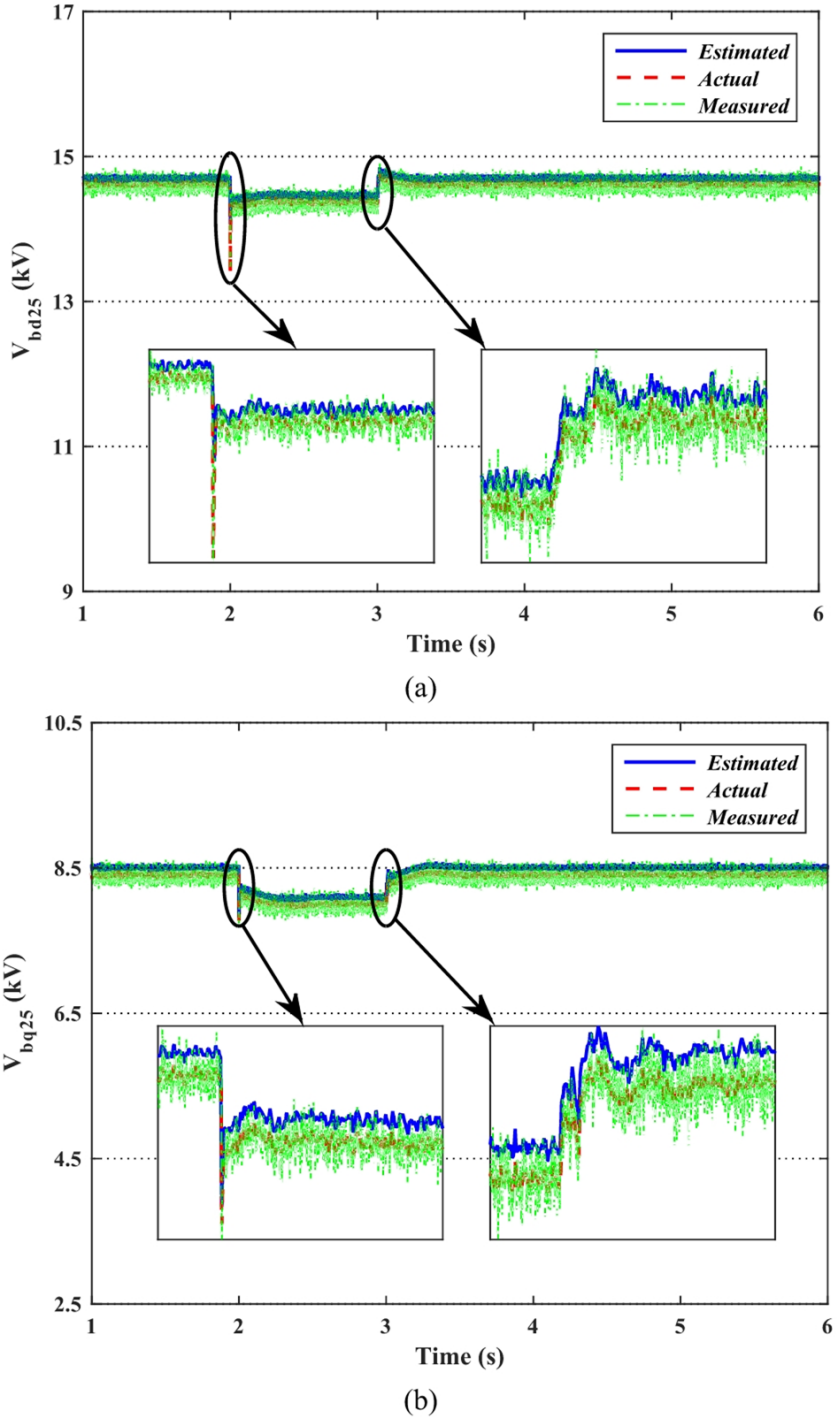
The worst-case scenario in DSE execution of Case *I*. (**a**) Voltage of bus-25 in d-axis (**b**) Voltage of bus-25 in q-axis.

**Fig. 13:**
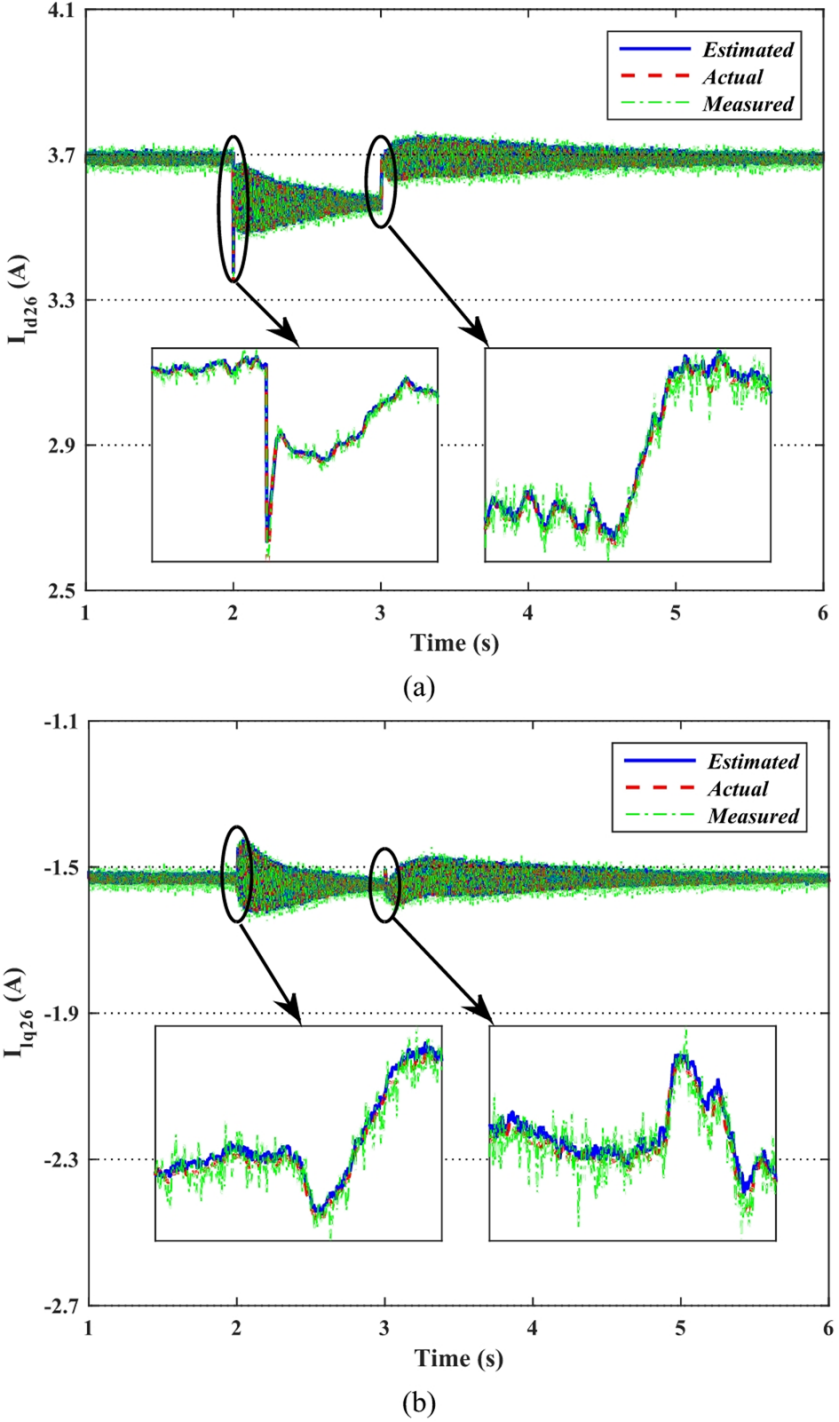
Sample of DSE results in Case *I*. (**a**) Load current of bus-26 in d-axis, (**b**) Load current of bus-26 in q-axis.

The voltage amplitude details for this bus on the *d* and *q* axes are illustrated in Fig. 12 under three conditions: actual, measured, and estimated. The actual values are derived from the IMG simulation output. The measured values are generated by adding noise to the actual values with a signal-to-noise ratio (SNR) of 40 dB. The estimated values are obtained from the output of the KF. The proximity of all three values indicates that the proposed method has provided a good estimation of the values, even in the presence of noise. The magnifications shown in the figure also confirm that the KF works well in the dynamic behavior of the IMG. As another example of DSE results, the load current of bus-26 up to 6 s in the *dq* frame is shown in Fig. 13.

In order to probabilistic analysis of line impedance in the execution of DSE, the material of the distribution lines is considered to be aluminum wire, and the temperature coefficient of the specific resistance of aluminum is equal to 0.0039 $\:1/^\circ\:\mathrm{C}$. The considered probability distribution of temperature changes in different conditions is normal with a mean of 20 °C and a standard deviation of 25 °C. The simulation of probabilistic analysis of line impedance in this case has been implemented using 100 MCS iterations, and the simulations have been conducted over 6 s.

**Fig. 14:**
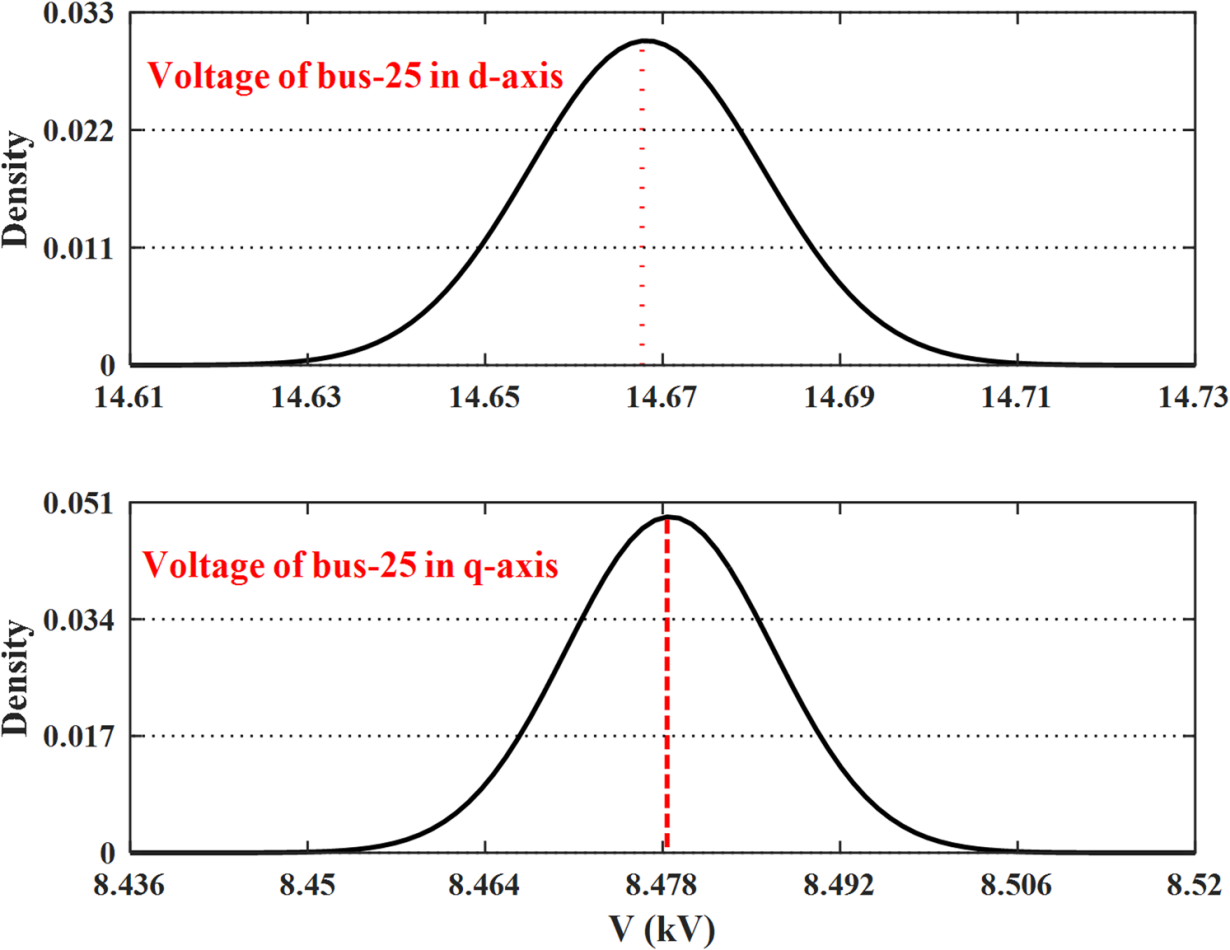
Probabilistic analysis of line impedance in DSE at second 3.

The influence of temperature on DSE is first analyzed independently across different seasons, excluding the effects of missing data. Subsequently, based on these analyses, a specific operating point is determined and applied for real-time monitoring and for addressing missing data scenarios. This methodology guarantees that the estimation framework is rigorously calibrated and optimized, accounting for temperature variations and data incompleteness. The voltage of bus 25 at the third second of DSE is shown as a normal distribution in the *dq* frame in Fig. 14, highlighting the probabilistic analysis of line impedance.

As shown in Fig. 14, at a temperature of 20 °C, which corresponds to the mean (peak) of the normal distribution curve, the *d*-axis voltage is measured at 14.6677 kV, while the *q*-axis voltage is 8.4783 kV. From these components, the phase voltage is calculated to be $\:\sqrt{{14.6677}^{2}+{8.4783}^{2}}=16.9418$ kV, with an RMS value (obtained by dividing by $\:\sqrt{2}$) of approximately 11.98 kV. Considering the nominal voltage rating of 12.66 kV, the per-unit (pu) voltage at 20 °C is therefore determined to be 0.94 pu. This normalized value provides a convenient basis for comparing voltage levels across different operating conditions and facilitates further analysis in system performance evaluation.

### Case II

This section aims to compensate for missing PMU data using a deep-trained structure. The GAIN network has been used to compensate for lost data. To train the GAIN module, the data is divided into two parts. The first 70% of the data, corresponding to the interval from 1 to 4.5 s, is used for network training. The next 5% (from 4.5 to 4.75 s) is allocated for validation, and the remaining 25% (from 4.75 to 6 s) is reserved for testing. Considering that in the study of the second scenario of load changes, two overloads have been applied in the last seconds, a proper evaluation of the network’s performance can be conducted. The simulation of the deep network has been implemented in the Colab environment with Python.

The generalization capability of the GAIN model is rigorously evaluated beyond the initial training set. Although training utilized data from $\:t\in\:[1,\:4.5]$ s covering nominal load variations, the testing interval $\:t\in\:[4.75,\:6]$ s exclusively contained two severe load increase events unseen during training, forcing the model to extrapolate to significantly different operating profiles. Furthermore, generalization was assessed across 15 distinct PMU loss scenarios (single and dual outages), demonstrating robustness to measurement anomalies that fundamentally alter the input distribution. Crucially, in real-time operation, GAIN continuously leverages the instantaneous multivariate statistical distribution of the available PMUs to impute missing data. This real-time adaptation allows the model to naturally track operating conditions deviating from the training set.

In addition to the previous modification in Case *I*, an additional load of 0.3 MW and 0.1 MVAr is connected to bus-19 at t = 4.9 s and disconnected at $\:t=5.65$ seconds. Similarly, the load change on bus-24 matches the changes on bus-19, except that this load is connected to the power grid at $\:t=5.25$ seconds and disconnected at $\:t=5.65$ seconds due to technical reasons. This scenario is designed to further evaluate changes in IMG dynamics using the proposed hybrid model. Overloads have been added to the IMG to evaluate the efficiency of the combined model of the KF and the GAIN deep network.

To evaluate PMU missing data and assess sensitivity of outputs to PMU loss, data unavailability is assumed over the final 1.25 s of the record. Fifteen scenarios are examined in two groups: (i) loss of a single PMU (five scenarios) and (ii) simultaneous loss of two PMUs (ten pairwise scenarios). “PMU loss” means all voltage and current measurements from that device to the EMS are unavailable; no information from that meter is received during the outage. Estimation error under various PMU loss scenarios is directly attributed to the degradation of measurement redundancy and network observability. The loss of a PMU, especially one located at a topologically critical bus (high connectivity or power exchange), significantly weakens the statistical constraints imposed on the estimator, forcing greater reliance on model dynamics and increasing uncertainty propagation. Furthermore, the efficacy of the GAIN imputation layer is governed by the instantaneous statistical coupling between the remaining and lost PMUs. Dual PMU outages compound this effect by simultaneously reducing spatial coverage and measurement redundancy, resulting in proportionally higher estimation uncertainty, even within the robust GAIN–KF framework.

**Fig. 15:**
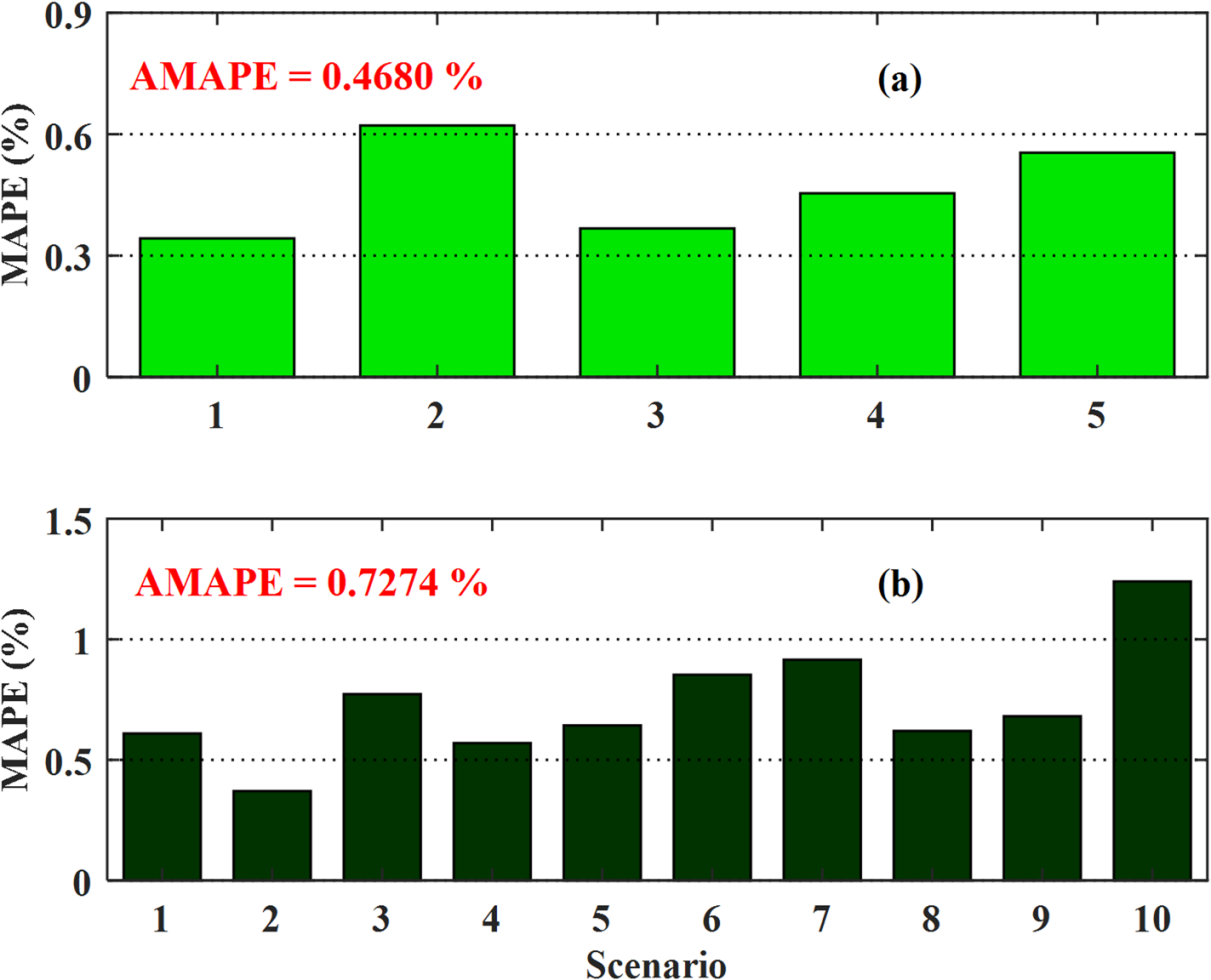
Missing data scenarios in Case *II*. (**a**) Scenarios of 1 PMU removal, (**b**) Scenarios of 2 PMU removal.

In the previous section, the buses at the end of the line were analyzed. Now, in this case, the average MAPE (AMAPE) index of the four buses is calculated to provide an overview of the data loss performance of the PMUs. This index will be defined and described as Eq. (24).

24 $$\:AMAPE=\frac{1}{N}\left(\sum\:_{\zeta\:=1}^{N}{MAPE}_{\zeta\:}\right)\times\:100\:\:\:$$

where $\:N$ represents the number of evaluated buses and $\:\zeta\:$ is the counter of these buses. The index value for each scenario, based on the evaluation of the four terminal buses in the test case, is illustrated in Fig. 15. In part (a), the first to fifth modes represent the individual failures of PMUs according to the bus numbers on which they are located. In part (b), the first to tenth modes indicate the failure of PMUs two by two in terms of bus number.

**Fig. 16:**
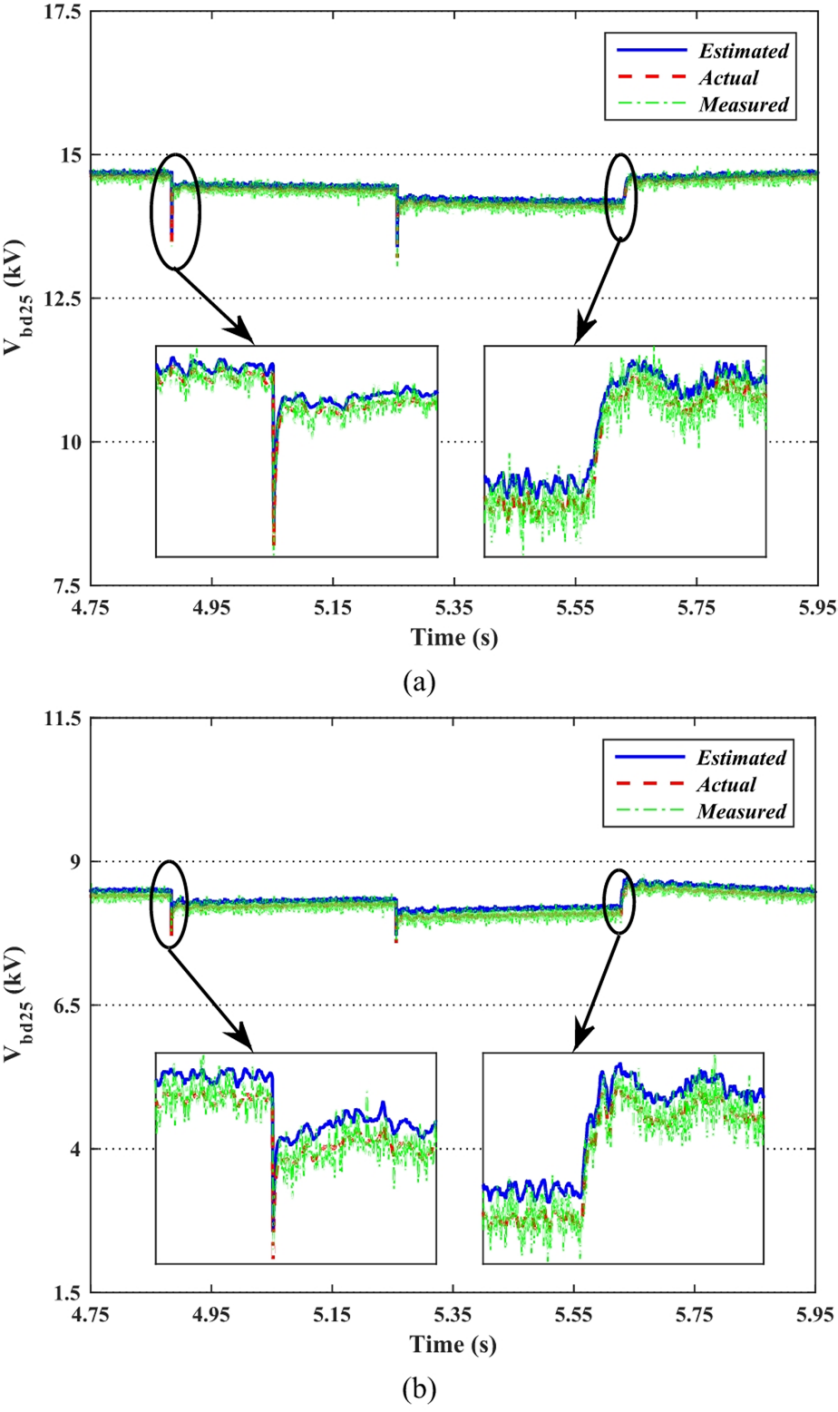
Sample of the sixth scenario in Case II. (**a**) Voltage of bus-25 in d-axis (**b**) Voltage of bus-25 in q-axis.

Examining the missing data percentage of a single PMU reveals that approximately 20% of the measured data is lost in these scenarios. When considering the missing data from both PMUs, it becomes evident that nearly 40% of the measured data is lost across these scenarios. To verify the performance of the proposed method in Case II, the voltage of bus-25 is shown in Fig. 16.

Based on the information presented in Table 4, both AE and VAE models were implemented using the PyTorch framework and trained under identical conditions to ensure a fair comparison. The Adam optimizer with a learning rate of 0.001 was employed for both models over 1000 training epochs. The AE model minimized the MSE, while the VAE incorporated an additional Kullback–Leibler divergence term to enforce a probabilistic latent space. Root mean square error (RMSE) was used as the evaluation metric for both models. These consistent settings established a controlled environment for evaluating and comparing the effectiveness of each model in imputing missing data.

**Fig. 17:**
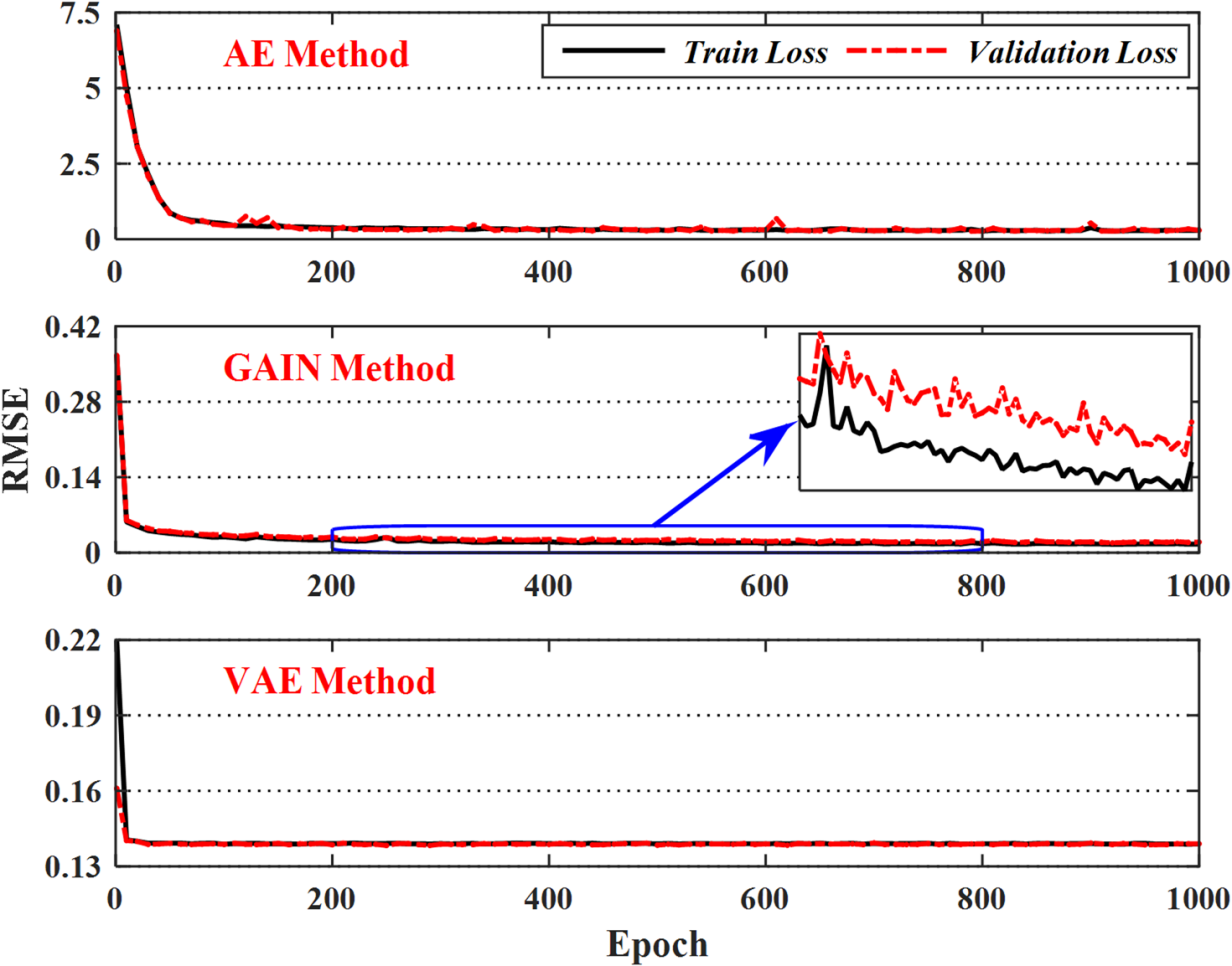
Convergence behavior of the loss function for GAIN, AE, and VAE methods.

Figure 17 illustrates a comparative analysis of the loss behavior across the three imputation methods: GAIN, AE, and VAE. The loss function in each case is evaluated based on the RMSE, providing a quantitative measure of reconstruction accuracy during the training process. As shown, all three models demonstrate stable convergence without signs of overfitting, indicating that they have not merely memorized the training data. In fact, the smooth and monotonic reduction in loss indicates proper convergence without oscillations or divergence. Instead, they have successfully learned to generalize from real data patterns to effectively reconstruct missing values. This performance reinforces the reliability of these models—particularly GAIN—in handling incomplete data in DSE tasks.

Overfitting is empirically ruled out by the results shown in Fig. 17, where the training and validation RMSE curves track closely, indicating learned generalizable relationships. Finally, the hybrid structure ensures physical consistency: GAIN only handles imputation, while the core SE relies on the physically consistent dq-domain dynamic model governed by the KF. These factors collectively confirm that GAIN generalizes effectively to novel and stressed IMG conditions.

By comparing the test results of the three methods—AE, VAE, and GAIN—AE recorded an error of 0.35872, VAE achieved 0.20344, while GAIN attained a significantly lower error of 0.02705. These results indicate that the GAIN model substantially outperforms the other two methods, demonstrating superior accuracy and effectiveness in imputing missing data or estimating dynamic states. In summary, a lower error value corresponds to better model performance, establishing GAIN as the most reliable approach among those tested.

To further position the proposed framework within state-of-the-art linear DSE techniques, a quantitative comparison between the KF and the H∞ filter is conducted under identical simulation conditions. Table 9 summarizes the estimation accuracy and computational performance of both filters across varying degrees of missing data. The results indicate that the KF achieves a lower estimation error, particularly under assumed Gaussian noise characteristics, while maintaining a significantly lower computational time per estimation step. Although the H∞​ filter enhances robustness against worst-case model uncertainties, it necessarily introduces additional matrix computations and conservatism constraints, resulting in a higher overall computational burden. These findings confirm that, for real-time DSE applications in IMGs with similar dynamic time constants, the KF offers a more favorable trade-off between estimation accuracy and computational efficiency.

**Table 9:** Quantitative comparison of linear DSE filters.

Metric	Kalman Filter (KF)	H∞ Filter
Average Estimation Error (20% missing data)	0.46%	0.63%
Average Estimation Error (40% missing data)	0.72%	0.91%
Mean Computational Time per Estimation Step	11.4 ms	17.8 ms

Quantitative results (Table 9) confirm this efficiency: the KF achieves accurate estimation even with 40% missing data in just 11.4 ms per step, making it computationally superior to alternatives like the H∞​ Filter (17.8 ms). This hybrid approach guarantees immediate data recovery while maintaining estimation within strict real-time constraints for IMG deployment.

The proposed hybrid KF–GAIN estimator is scalable and generalizable to larger and more diverse IMGs. The framework is inherently measurement-driven and modular, where the GAIN component exploits multivariate statistical dependencies among available PMU measurements to reconstruct missing data, and these dependencies typically become richer with increasing system size or structural diversity. Similarly, the *dq*-domain KF operates at the device level and scales in a predictable manner as additional DGs, loads, or buses are incorporated, without requiring modifications to the estimation structure. Although the numerical validation is conducted on a compact microgrid for clarity and controlled analysis, neither component of the proposed framework is restricted to the specific configuration of the test system. Consequently, in larger and more heterogeneous IMGs, the increased availability of PMU measurements can provide greater statistical redundancy, which is expected to further enhance real-time SE accuracy.

## Conclusion

Due to the critical role of dynamic state estimation (DSE) in islanded microgrids (IMGs), and the inherent uncertainties caused by line impedance variations and missing data, this study proposed a linear hybrid framework that integrates the Kalman Filter (KF) with a deep learning model known as GAIN. The objective was to enhance the accuracy and robustness of DSE by simultaneously addressing data incompleteness and the probabilistic nature of temperature-dependent line impedance. A representative IMG was simulated with a gradually increasing load profile at one-second intervals. Initially, DSE was conducted using the KF under the assumption of complete data availability, and the estimation performance was evaluated using MSE and MAPE across four terminal buses. The impact of probabilistic variations in line impedance, driven by ambient temperature fluctuations, was also incorporated into the analysis. To handle missing data from PMUs, the GAIN model was employed for data imputation. Fifteen test scenarios were considered, in which 10 and 20 out of 48 total input signals were randomly removed, corresponding to the loss of one and two PMU measurements, respectively. The KF was then used to perform DSE on the reconstructed data, with the average MAPE (AMAPE) used as the primary evaluation metric. Comparative results with baseline models—autoencoder (AE) and variational autoencoder (VAE)—highlight the effectiveness of GAIN. Hyperparameters (GAIN, AE, VAE) were grid-searched, and robustness was assessed via single- and pairwise-PMU sensitivity. GAIN achieved a significantly lower imputation error of 0.02705, compared to 0.35872 for AE and 0.20344 for VAE, demonstrating its superior performance in reconstructing incomplete datasets. Furthermore, the analysis showed that the KF maintained strong performance even in the presence of data loss: with 20% of input data missing, the KF produced an average error of 0.46%, and with 40% data loss, the error remained within 0.72%. Overall, the proposed KF-GAIN framework demonstrates high potential for reliable DSE in IMGs, particularly under realistic operating conditions with uncertain line parameters and partial measurement availability. The scalability of the KF–GAIN framework has been analytically discussed in this paper. Further investigations on large-scale benchmark systems and real-world distribution networks could provide additional insight into its performance under more complex operating conditions. Such extensions are considered promising directions for future research.

## Data Availability

The datasets used and/or analyzed during the current study available from the corresponding author on reasonable request.

## Abbreviations

IMG: Islanded microgrid
SE: State estimation
SSE: Static state estimation
DSE: Dynamic state estimation
EMS: Energy management system
DG: Distributed generation
VSI: Voltage source inverter
PMU: Phasor measurement unit
KF: Kalman filter
UKF: Unscented Kalman filter
EKF: Extended Kalman filter
WLS: Weighted least squares
PF: Particle filter
dq: Direct and quadrature
GAN: Generative adversarial network
GAIN: Generative adversarial imputation network
MCS: Monte Carlo simulation
PI: Proportional integral
PWM: Pulse width modulation
BSE: Bayesian state estimation
MSE: Mean squared error
RMSE: Root mean squared error
MAPE: Mean absolute percentage error
AMAPE: Average mean absolute percentage error
AE: Autoencoder
VAE: Variational autoencoder

## Parameters/variables

$$\omega$$: Angular frequency
${\omega}_{c}$: Common rotating frequency
$P$*,*$Q$: Active and reactive power
${V}_{o}^{RLC}$: Output voltage of the RLC filter
${v}_{od}$: Output voltage of RLC filter in d-axis
${v}_{oq}$: Output voltage of the RLC filter in q-axis
${I}_{o}^{RLC}$: Output current of the RLC filter
${\omega}_{cut}$: Cutoff frequency of the low-pass filter
${i}_{od}$: Output current of the RLC filter in d-axis
${i}_{oq}$: Output current of the RLC filter in q-axis
$${\omega}_{r}$$: Rated frequency
$${V}_{r}$$: Rated voltage
$${\omega}_{o}^{pc}$$: Inverter frequency
${V}_{o}^{pc}$: Output voltage of the power controller
${V}_{od}^{pc}$: Output voltage of power controller in d-axis
${V}_{oq}^{pc}$: Output voltage of power controller in q-axis
${M}_{p}$: Active power droop coefficient
${N}_{q}$: Reactive power droop coefficient
${I}_{od}^{vc}$: Output current of the voltage controller in d-axis
${I}_{oq}^{vc}$: Output current of the voltage controller in q-axis
${V}_{od}^{cc}$: Output voltage of the current controller in the d-axis
${V}_{oq}^{cc}$: Output voltage of the current controller in the q-axis
${I}_{od}^{ub}$: Inverter output current in the d-axis
${I}_{oq}^{ub}$: Inverter output current in the q-axis
${I}_{l{d}_{i}}, {I}_{l{q}_{i}}$: d- and q-axis components of the load current at bus *i*.
${I}_{o{d}_{i}}, {I}_{o{q}_{i}}$: Output current components of the converter at bus *i*.
${I}_{b{d}_{ij}},{I}_{b{q}_{ij}}$: Line current components in dq-axes between buses *i* and *j*.
${V}_{bd,i},{V}_{bq,i}$: Voltage components of bus *i* in the d- and q-axes.
${V}_{bd,j},{V}_{bq,j}$: Voltage components of bus *j* in the d- and q-axes.
${R}_{o}$: Resistance at reference temperature
$$z$$: Impedance
${K}_{{P}_{vc}}, {K}_{{I}_{vc}}$: Proportional and integral gains of voltage PI controller
${K}_{{P}_{cc}}, {K}_{{I}_{cc}}$: Proportional and integral gains of current PI controller
${R}_{{l}_{i}}, {L}_{{l}_{i}}$: Resistance and inductance of load connected to bus *i*.
${R}_{{b}_{ij}}, {L}_{{b}_{ij}}$: Resistance and inductance of line connecting buses *i* and *j*.
${R}_{n}$: Large virtual grounding resistor
$\sum{I}_{{bd}_{i\left(in\right)}}$: SUM of incoming and outgoing d-axis branch currents at bus *i*.
$\sum{I}_{{bd}_{i\left(out\right)}}$: Sum of incoming and outgoing q-axis branch currents at bus *i*.
$A$: System dynamic matrix
$B$: Input control matrix
$C$: Measurement transformation matrix
$D$: Input control matrix in measurement
$v(t)$: Measurement noise vector at time *t*
$X(t)$: System state vector at time *t*
$Y(t)$: Measurement vector at time *t*
$u(t)$: Input control vector at time *t*
$w(t)$: Process noise vector at time *t*
$K$: Kalman filter gain matrix
$\mathcal{R}$: Measurement noise covariance matrix
$\mathcal{M}$: Missing data locations
$$\mathcal{Z}$$: Noise
$${\mathbb{G}}$$: Generator
$${\mathbb{D}}$$: Discriminator
$${\mathbb{I}}$$: Identity matrix
$${X}_{k}$$: Missing data
${\widetilde{X}}_{k}$: Reconstructed missing data
$$⨀$$: Hadamard product
$\alpha$: Temperature coefficient of resistivity
$$\Delta T$$: Temperature variation
$\mu$: Mean ambient temperature
$\sigma$: Standard deviation of ambient temperature
$$n$$: Sample size
$${y}_{act}$$: Actual values
$${y}_{est}$$: Estimated values
${R}_{RL}, {L}_{RL}$: RL filter resistance and inductance in VSI model
${C}_{RLC}, {L}_{RLC}$: RLC filter capacitor and inductor in VSI model
